# Brain cortical alterations in COVID-19 patients with neurological symptoms

**DOI:** 10.3389/fnins.2022.992165

**Published:** 2022-10-20

**Authors:** Gretel Sanabria-Diaz, Manina Maja Etter, Lester Melie-Garcia, Johanna M. Lieb, Marios-Nikos Psychogios, Gregor Hutter, Cristina Granziera

**Affiliations:** ^1^Translational Imaging in Neurology (ThINK) Basel, Department of Biomedical Engineering, Faculty of Medicine, University Hospital Basel, University of Basel, Basel, Switzerland; ^2^Department of Neurology, University Hospital Basel, Basel, Switzerland; ^3^Research Center for Clinical Neuroimmunology and Neuroscience Basel (RC2NB), University Hospital Basel, University of Basel, Basel, Switzerland; ^4^Brain Tumor Immunotherapy Lab, Department of Biomedicine, University Hospital Basel, University of Basel, Basel, Switzerland; ^5^Division of Neurosurgery, University Hospital Basel, Basel, Switzerland; ^6^Department of Neuroradiology, Clinic of Radiology and Nuclear Medicine, University Hospital Basel, Basel, Switzerland

**Keywords:** SARS-CoV-2, COVID-19, gray matter volume (GMV), cortical thickness, surface area, neurological symptoms

## Abstract

**Background:**

Growing evidence suggests that the central nervous system is affected by severe acute respiratory syndrome coronavirus 2 (SARS-CoV-2), since infected patients suffer from acute and long-term neurological sequelae. Nevertheless, it is currently unknown whether the virus affects the brain cortex. The purpose of this study was to assess the cortical gray matter volume, the cortical thickness, and the cortical surface area in a group of SARS-CoV-2 infected patients with neurological symptoms compared to healthy control subjects. Additionally, we analyzed the cortical features and the association with inflammatory biomarkers in the cerebrospinal fluid (CSF) and plasma.

**Materials and methods:**

Thirty-three patients were selected from a prospective cross-sectional study cohort during the ongoing pandemic (August 2020–April 2021) at the university hospitals of Basel and Zurich (Switzerland). The group included patients with different neurological symptom severity (Class I: nearly asymptomatic/mild symptoms, II: moderate symptoms, III: severe symptoms). Thirty-three healthy age and sex-matched subjects that underwent the same MRI protocol served as controls. For each anatomical T1w MPRAGE image, regional cortical gray matter volume, thickness, and surface area were computed with FreeSurfer. Using a linear regression model, cortical measures were compared between groups (patients vs. controls; Class I vs. II–III), with age, sex, MRI magnetic field strength, and total intracranial volume/mean thickness/total surface area as covariates. In a subgroup of patients, the association between cortical features and clinical parameters was assessed using partial correlation adjusting for the same covariates. *P*-values were corrected using a false discovery rate (FDR).

**Results:**

Our findings revealed a lower cortical volume in COVID-19 patients’ orbitofrontal, frontal, and cingulate regions than in controls (*p* < 0.05). Regional gray matter volume and thickness decreases were negatively associated with CSF total protein levels, the CSF/blood-albumin ratio, and CSF EN-RAGE levels.

**Conclusion:**

Our data suggest that viral-triggered inflammation leads to neurotoxic damage in some cortical areas during the acute phase of a COVID-19 infection in patients with neurological symptoms.

## Introduction

The severe acute respiratory syndrome coronavirus 2 (SARS-CoV-2) continues to affect millions of people worldwide. After 2 years of the pandemic, current evidence suggests that the virus directly or indirectly impacts the brain (Kremer et al., 2020; Ladopoulos et al., 2021; Manca et al., 2021; Pajo et al., 2021). Frequently, patients who suffer from a mild to a severe infection show neurological manifestations (García-Azorín et al., 2021; Meppiel et al., 2021; Taquet et al., 2021), which are described in 30–80% of hospitalized patients (Helms et al., 2020; Kotfis et al., 2020).

However, the underlying mechanisms leading to brain alterations and cognitive impairment remain unknown. There is some evidence pointing to a viral neurotropism (Paterson et al., 2020; de Erausquin et al., 2021), virus-induced inflammatory state (refereed as a cytokine storm) (Deleidi and Isacson, 2012; Butowt et al., 2021; Yang et al., 2021) and systemic post-infectious inflammation (McQuaid et al., 2021). Nevertheless, the virus’ presence in the human brain has still to be demonstrated (von Weyhern et al., 2020; Brady et al., 2021; McQuaid et al., 2021).

Previous studies during a SARS-CoV-2 chronic phases and after infection revealed an increase in gray matter volume (GMV) in brain regions (Lu et al., 2020) and the frontotemporal network (Duan et al., 2021). Regarding SARS-CoV-2-infected patients with neurological symptoms, it has been reported that cortical thickness (CTh) decreases in frontal and limbic areas (Qin et al., 2021). Furthermore, a recent longitudinal population study using pre- and post-COVID MRI scans from 401 SARS-CoV-2 -infected patients and 384 healthy controls reported cortical thickness reduction in the orbitofrontal cortex and parahippocampal gyrus, more pronounced gray matter tissue damage in regions that were functionally connected to the primary olfactory cortex, and a greater reduction in global brain size after SARS-CoV-2 infection (Douaud et al., 2021). Nevertheless, this study mainly focused on patients with mild infection (Douaud et al., 2021). These findings suggest a link between SARS-CoV-2 infection and brain morphometric alterations. However, they applied a single morphometric approach based on GMV or CTh analysis in mildly affected patients without neurological complications during the post-infection phase.

Instead, in this study, we investigated the characteristics of the cortex of COVID-19 patients depicting different severity stages of neurological symptoms and compared the findings to healthy subjects by using a multi-morphometric approach. It is well-documented that CTh, surface area (SA), and GMV capture different underlying morphological processes (Dickerson et al., 2009; Panizzon et al., 2009; Lemaitre et al., 2012). The implementation of this approach may offer insights into which feature is more pertinent to detecting cortical alterations in neurologically compromised patients. Additionally, we investigated the association between the morphometric measures in COVID-19 patients with neurological symptoms and body fluids measures related to (i) infection, (ii) organ damage, and (iii) concomitant hyperinflammatory response.

## Materials and methods

### Participants

Data were obtained from a prospective, two-center, cross-sectional study (clinicaltrials.gov NCT04472013, IRB approval EKNZ 2020-01503) including COVID-19 patients from August 2020 to April 2021 at the Swiss University Hospitals of Basel and Zurich. Participants remained anonymous, and written consent was given by the patients or a legal representative. All clinical investigations were conducted according to the principles expressed in the Declaration of Helsinki. The study included a neurological examination, lumbar puncture, blood withdrawal for cerebrospinal fluid (CSF) and plasma soluble protein analysis, cranial MRI, or CT scan. Neurological examination was performed using the National Institutes of Health Stroke Scale (NIHSS). Glasgow Coma Scale (GCS) was used in sedated patients.

Thirty-two patients underwent contrast-enhanced brain MRI imaging. Due to logistic challenges, staffing, and medical surveillance issues during the COVID-19 pandemic, five patients underwent cranial computed tomography (CT) instead of brain MRI. In contrast, one patient was imaged with a brain MRI and cranial CT.

For this study, the SARS-CoV-2 infection cohort was selected retrospectively based on the following inclusion criteria: (1) age > 18 years, (2) SARS-CoV-2 infection confirmed by reverse transcriptase PCR (rRT-PCR) testing, and (3) a 3D high-resolution T1-weighted MRI sequence of the whole brain at the time of their positive SARS-CoV-2 qRT-PCR test (Table 1). The exclusion criteria were (1) SARS-CoV-2 RT-PCR-negative testing and (2) pregnancy. Retrospectively, biobanked age- and sex-matched healthy individuals (*n* = 33) with no neurological pre-existing risk factor other than headache served as controls (Table 1).

**TABLE 1:** Demographics, clinical, and paraclinical characteristics of COVID-19 patients and the control group.

	Patients	Controls	*p*-value
Numbers of subjects	33	33	na
Age	50.45 ± 19.13	51.64 ± 23.25	0.78
Sex, Male/Female (M/F)	13/20	12/21	0.80
Neuro-COVID class	I (*n* = 15), II (*n* = 8), III (*n* = 5)	na	na
CSF leukocytes (mmol/l)	*n* = 16 (4.38 ± 3.83)	na	na
CSF lactate (mmol/l)	*n* = 15 (1.89 ± 0.52)	na	na
CSF protein total (mmol/l)	*n* = 16 (345.41 ± 186.37)	na	na
CSF blood albumin ratio	*n* = 14 (6.50 ± 4.61)	na	na
CSF glucose (mmol/l)	*n* = 15 (4.54 ± 1.33)	na	na
Plasma TRANCE (pg/mL)	*n* = 20 (2.74 ± 0.93)	na	na
Plasma EN-RAGE (pg/mL)	*n* = 20 (3.30 ± 1.37)	na	na
CSF OPG (pg/mL)	*n* = 18 (9.55 ± 0.68)	na	na
CSF TRANCE (pg/mL)	*n* = 18 (-0.31 ± 0.29)	na	na
CSF EN-RAGE (pg/mL)	*n* = 18 (0.43 ± 0.74)	na	na
Weight (kg)	69.06 ± 11.62	72.79 ± 15.85	0.44
Height (meters)	1.70 ± 0.10	1.69 ± 0.10	0.73
MRI magnetic field strength	1.5 T (*n* = 29) 3 T (*n* = 4)	1.5 T (*n* = 11) 3 T (*n* = 22)	na
TIV (cm^3^)	1520.03 ± 179.37	1523.14 ± 146.27	0.86
Mean cortical thickness (mm^3^)	2.34 ± 0.15	2.39 ± 0.16	0.27
Surface area (mm^2^)	1596.4 ± 198.25	1655.79 ± 163.27	0.10

Data are shown as Mean ± SD and/or n (sample size). *P*-values are obtained by independent-test, Pearson chi^2^, and Non-parametric Mann-Whitney *U* test when a variable is not normally distributed (Shapiro-Wilk *P* < 0.05), or there is no homogeneity of variances (Levene’s test). n, sample size; sd, standard deviation; CSF, Cerebrospinal fluid; TIV, Total Intracranial Volume; TRANCE, tumor necrosis factor-related activation-induced cytokine; EN-RAGE, receptor for advanced glycation end-products binding protein; OPG, osteoprotegerin; na, not applicable; cm, centimeters; mm, millimeters; kg, kilograms; mmol/l, millimole per liters; pg/mL, picograms per milliliter; M, male; F, female; I, NeuroCOVID Class 1; II, NeuroCOVID Class 2; III, NeuroCOVID Class 3.

Additional information about pre-existing risk factors and clinical indications for the brain MRI study of patients can be found in Supplementary Tables 2, 3. Patients with strokes were not included in our sample. As expected, several comorbidities characterized the group of hospitalized SARS-CoV-2 infected patients.

Enrolled patients (*n* = 33) were subdivided into three Neuro-COVID severity classes, referred to as Class I (*n* = 16), II (*n* = 10), or III (*n* = 7). The neurological symptoms in Class I involved mild signs/symptoms such as anosmia, ageusia, headache, and dizziness. Patients in Class II showed moderate signs/symptoms (e.g., mono/para/quadriparesis, fatigue), whereas Class III represented those with severe signs/symptoms such as seizures, and cognitive impairment (Fotuhi et al., 2020; Etter et al., 2022). Patients with pre-existing illnesses in their past medical history were included. Based on the sample size per Class, we considered the following groups for further analyses (Table 2).

**TABLE 2:** Demographics, clinical, and paraclinical characteristics of Neuro-COVID Class I and Class II–III groups.

	Class I	Class II–III	*p*-value
Numbers of subjects	16	17	na
Age	47.87 ± 21.27	47.22 ± 15.22	0.96
Sex, male/female (M/F)	6/9	4/9	0.61
CSF leukocytes (mmol/l)	n = 8 (3.63 ± 3.54)	*n* = 6 (4 ± 2.53)	0.65
CSF lactate (mmol/l)	*n* = 9 (1.68 ± 0.21)	*n* = 6 (1.86 ± 0.53)	0.72
CSF protein total (mmol/l)	*n* = 9 (293.56 ± 80.53)	*n* = 6 (354.50 ± 188.20)	0.40
CSF blood albumin ratio	*n* = 9 (5.03 ± 1.84)	*n* = 4 (7.20 ± 4.73)	0.60
CSF glucose (mmol/l)	*n* = 9 (4.21 ± 1.33)	*n* = 6 (4.70 ± 1.50)	0.55
Plasma TRANCE (pg/mL)	*n* = 11 (3.32 ± 0.75)	*n* = 7 (2.06 ± 0.55)	**0.001**
Plasma EN-RAGE (pg/mL)	*n* = 11 (2.65 ± 1.39)	*n* = 7 (4.12 ± 0.98)	**0.003**
CSF OPG (pg/mL)	*n* = 10 (9.31 ± 0.64)	*n* = 6 (9.79 ± 0.68)	0.18
CSF TRANCE (pg/mL)	*n* = 10 (-0.39 ± 0.31)	*n* = 6 (-0.27 ± 0.26)	0.47
CSF EN-RAGE (pg/mL)	*n* = 10 (0.33 ± 0.26)	*n* = 6 (0.12 ± 0.42)	0.22
Weight (kg)	64.73 ± 10.66	69.92 ± 7.45	0.22
Height (meters)	1.70 ± 0.09	1.68 ± 0.09	0.42
MRI magnetic field strength	1.5 T (*n* = 15)	1.5 T (*n* = 9) 3 T (*n* = 4)	**0.02**
TIV (cm^3^)	1516.44 ± 160.10	1478.65 ± 195.50	0.58
Mean cortical thickness (mm^3^)	2.37 ± 0.13	2.36 ± 0.17	0.88
Surface area (mm^2^)	1650.3 ± 161.20	1592.94 ± 244.39	0.46

Data are shown as Mean ± SD and/or n (sample size). *P*-values were obtained by independent-test, Pearson chi^2^, and Non-parametric Mann-Whitney *U* test when a variable was not normally distributed (Shapiro-Wilk *P* < 0.05), or there was no homogeneity of variances (Levene’s test). n, sample size; sd, standard deviation; CSF, Cerebrospinal fluid; TIV, Total Intracranial Volume; TRANCE, tumor necrosis factor-related activation-induced cytokine; EN-RAGE, receptor for advanced glycation end-products binding protein; OPG, osteoprotegerin; na, not applicable; cm, centimeters; mm, millimeters; kg, kilograms; mmol/l, millimole per liters; pg/mL, picograms per milliliter; M, male; F, female; I, Neuro-COVID Class 1; II, Neuro-COVID Class 2; III, Neuro-COVID Class 3. Bold indicates *p*-values < 0.05.

### Biomarker measurements in cerebrospinal fluid

In a subset of patients, CSF and blood examinations were performed simultaneously during the acute phase of COVID-19, on an average latency period of 3.5 days (range: 1–12 days) after the first positive SARS-CoV-2 qRT-PCR test result. Measures in the CSF included the number of leukocytes, levels of lactate and total protein, and CSF/blood-albumin-ratio (Gabay and Kushner, 2008). Additionally, chemokines, soluble cell membrane proteins, and cytokines, were measured using the Olink 96 target neurology^1^ and Olink 96 target inflammation^2^ panels. Based on previous reports of associations with a SARS-CoV-2 infection (Etter et al., 2022; Jarius et al., 2022), we selected five cytokines for our correlation analysis (plasma-TRANCE, plasma-EN-RAGE, CSF-OPG, CSF-TRANCE, and CSF-EN-RAGE). For further information about the CSF and blood-derived measures and their clinical significance, see Supplementary Table 1.

### Imaging protocols

3D high-resolution T1-weighted anatomical images were acquired using two different MRI scanners: Scanner 1: 1.5 Tesla Siemens Avanto Fit and Scanner 2: 3 Tesla Siemens Skyra. A Magnetization Prepared—RApid Gradient Echo (MPRAGE) pulse sequence covering the whole brain was used in both MRI scanners with the following parameters. Scanner 1: 160 contiguous slices of 1 mm thickness in sagittal orientation; in-plane FOV = 256 × 256 mm^2^, and matrix size 256 × 256 yielding an in-plane spatial resolution of 1 × 1 mm^2^ and voxel size of 1 × 1 × 1 mm^3^. The echo (TE), repetition (TR), and inversion (TI) times were set to TE/TR/TI = 2.8 ms/2,400 ms/900 ms with a flip angle FA = 8°. Scanner 2: A 160 contiguous slices of 1 mm thickness in sagittal orientation; in-plane FOV = 256 × 240 mm^2^, and matrix size 256 × 240 yielding an in-plane spatial resolution of 1 × 1 mm^2^ and voxel size of 1 × 1 × 1 mm^3^. The echo, repetition, and inversion times were set to TE/TR/TI = 2.98 ms/2,300 ms/900 ms with a flip angle FA = 9°.

### Imaging analysis

Cortical reconstruction and volumetric segmentation were performed with Freesurfer.^3^ The technical details of these procedures are described in prior publications (Dale and Sereno, 1993; Fischl et al., 1999, 2002, 2004; Fischl and Dale, 2000). Briefly, the preprocessing steps include motion correction, intensity normalization, Talairach registration, skull stripping, subcortical white matter segmentation, tessellation of the gray matter/white matter boundary, topology correction, surface deformation to end with a surface 3D model of the cortex by means of intensity and continuity information.

### Regional gray matter volume, cortical thickness, and surface area computations

Applying FreeSurfer’s pipeline, local CTh and SA were calculated at each vertex. Each cortical segmentation was visually checked for inaccuracies and manually corrected. CTh was calculate as the shortest distance between the GM/WM boundary and pial surface at each vertex across the cortical mantle, measured in millimeters (mm). GMV was calculated with FreeSurfer’s automated procedure for volumetric measures (Fischl et al., 2002, 2004).

In addition, global brain measures (mean CTh and total SA) were computed using the FreeSurfer (Fischl et al., 2002). The total intracranial volume (TIV) estimation was based on the sum of resulting raw values for gray matter, white matter, and CSF derived from the SPM 12 (Statistical Parametric Mapping) (Ashburner and Friston, 2005).

We selected 34 gyral-based regions of interest per hemisphere, according to the Desikan-Killiany atlas (Desikan et al., 2006). For each of the 68 bilateral cortical regions, FreeSurfer calculates (i) the average CTh (in mm), (ii) total cortical SA of the pial (in mm^2^), and (iii) the cortical GMV (in mm^3^).

### Statistical analysis

All variables’ normal distributions and equality of variances were assessed using Shapiro-Wilk tests and Levene’s tests. As appropriate, clinical and demographic variables were compared between groups with an independent *t*-test, Mann-Whitney, or Chi-square tests.

Regional morphometric measures were compared between groups (patients vs. controls) and Neuro-COVID Classes (Class I vs. Class II-III) using a linear regression model. The covariates were age, gender, age × gender interaction, MRI magnetic field strength, and global measures (TIV/mean CTh/total SA). False discovery rate (FDR) correction was used to adjust for multiple comparisons by the number of structures.

The association between morphometric brain descriptors and biological parameters was assessed using a partial correlation and adjusted for age, sex, age × sex interaction, MRI magnetic field strength, and global brain measure (TIV/mean CTh/total SA). The resulting values were corrected for multiple comparisons using FDR correction.

The statistical analysis was performed using the JASP^4^ and MATLAB software (‘‘partialcorri.m’’ function).^5^

## Results

### Sample description statistics

The groups did not significantly differ in age, gender, education, or global measures (TIV, Mean CTh, SA, Global gray matter, Global white matter, and total CSF) (Table 1). However, there was a trend in patients showing lower values in all global measures compared to controls (Table 1). Regarding the body-fluid measures, 25% of the patients showed increased leukocytes levels (4/16), 33% abnormal lactate levels (5/15), 12.5% increased protein levels (2/16), 46.7% increased glucose levels (7/15), and 21.4% an increased CSF/blood-albumin ratio (3/14).

The Neuro-COVID Class I and II-III did not differ significantly in age, gender, education, or global measures (TIV, Mean CTh, SA, Global gray matter, Global white matter, and total CSF) (Table 2). However, the Classes were statistically different in plasma TRANCE levels, plasma EN-RAGE levels, and MRI magnetic field applied to acquire 3D T1 images (all *p* < 0.05) (Table 2).

### Morphometric differences between the SARS-CoV-2 and controls group

Three cortical regions showed different GMV between SARS-CoV-2 infected patients and controls. Patients exhibited lower GMV values in the right rostral anterior cingulate (controls mean = 1.90; patients mean = 0.38; *p*-corrected = 0.04), left medial orbitofrontal (controls mean = 5.08; patients mean = 0.84; *p*-corrected = 0.04), and left superior frontal regions (controls mean = 22.01; patients means = 3.75; *p*-corrected = 0.04). There were no significant differences in CTh and SA between groups after FDR correction. However, patients showed a trend to lower values compared to the control group (see Supplementary Tables 4–6).

When the uncorrected *p*-values were analyzed, 12 regions were found to have lower GMV in COVID-19 patients, and 50% were located in frontal structures. The rest belonged to limbic, parietal, and temporal regions (Supplementary Table 5).

For a complete list of group contrast and regional *p*-values, see Supplementary Tables 4–6.

### Brain regional morphometric differences between the Class I and Class II–III patients

After multiple comparison corrections, the Neuro-COVID Class I and II-III did not significantly differ in GMV, CTh, and SA. However, several regions showed a GMV decrease in patients with moderate-severe neurological symptoms compared to the mild ones before FDR correction (posterior cingulate, rostral anterior cingulate, precuneus, inferior parietal, pars orbital, and middle temporal) (*p*-uncorrected < 0.05) (Supplementary Table 7). The same patterns were observed for CTh and SA in Class II-III compared to Class I (Supplementary Tables 7–9).

### Regional gray matter volume and cortical thickness associated with clinical variables in SARS-CoV-2 infected patients

In a subgroup of patients, we studied the association between regional GMV, CTh, and SA with CSF and blood measures linked to infectious and inflammatory processes (CSF leukocytes, CSF lactate, CSF total protein, CSF/blood-albumin-ratio, CSF/plasma EN-RAGE levels, CSF/plasma OPG levels, and CSF/plasma TRANCE levels).

**FIGURE 1:**
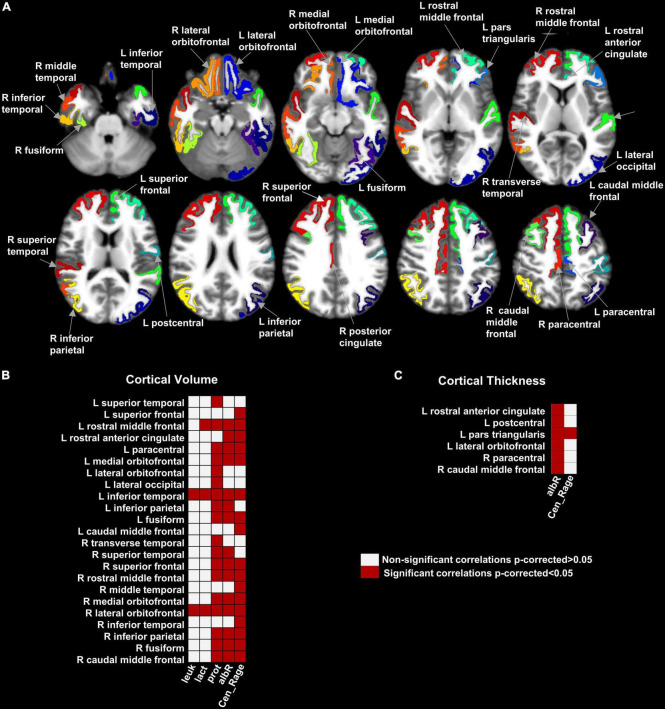
Map of the brain regions with a significant association between gray matter volume, cortical thickness, and clinical variables in hospitalized SARS-CoV-2 patients with neurological symptoms/signs. Panel **(A)** shows the 29 brain regions with significant correlation values for GMV and cortical thickness after multiple comparison corrections (False Discovery Rate- FDR). Panels **(B,C)** shows the matrices representing the association significance (significant *p*-corrected < 0.05 in red squares). CSF, Cerebrospinal fluid; leuk, leukocytes; lact, lactate; prot, protein; albR, Albumin CSF-blood ratio; Cen_Rage: CSF EN-RAGE, extracellular receptor for advanced glycation end-products binding protein; R, right; L, left.

Figure 1 shows 23 brain regions where GMV was negatively correlated with the CSF leukocyte count, CSF lactate, CSF total protein, CSF/blood-albumin-ratio, and CSF EN-RAGE levels after FDR correction (*p* < 0.05) (Supplementary Table 10). A higher CTh in six cortical regions (frontal, orbitofrontal, and temporal cortices) was found to be associated with higher CSF EN-RAGE levels and CSF/blood-albumin-ratio levels in all regions but one, where we measured a negative correlation (Figure 1 and Supplementary Table 11). SA did not correlate with any biological variable.

**FIGURE 2:**
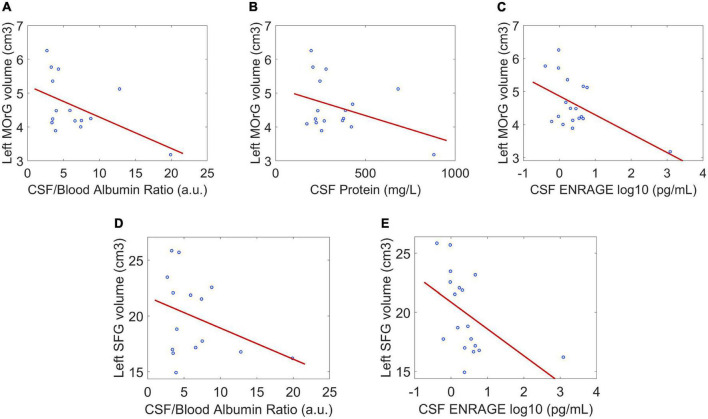
Correlation between regional gray matter volume and CSF clinical parameters in regions where gray matter volume between patients and controls was significantly different. **(A–C)** The negative associations between gray matter volume of the left medial orbitofrontal gyrus and CSF/blood Albumin Ratio, CSF protein, and CSF EN-RAGE. **(D,E)** The negative associations between gray matter volume of the left superior frontal gyrus and CSF/blood Albumin Ratio, CSF EN-RAGE. *P* < 0.05 is considered significant. Dot blue represents an individual patient’s measure. MOrG, medial orbitofrontal gyrus; SFG, superior frontal gyrus; cm^3^, centimeters cubic; a.u, arbitrary unit; pg/mL, picograms per milliliter.

Between the statistically significant associations, we found three regions previously reported with lower GMV in patients compared to controls: right rostral anterior cingulate, left medial orbitofrontal, and left superior frontal (subsection “Morphometric differences between the SARS-CoV-2 and controls group”). They were negatively correlated with the CSF/blood-albumin-ratio and CSF EN-RAGE levels in the patients subgroup with a CSF study (Figure 2).

## Discussion

In this study, SARS-CoV-2-infected patients with neurological symptoms ranging from mild to severe exhibited lower cortical volume in the orbitofrontal, frontal, and cingulate cortex compared to healthy controls. Besides, in those patients, a lower regional cortical GMV and CTh were associated with an increase in total protein CSF levels, CSF/blood-albumin ratio, and the levels of an inflammatory cytokine named EN-RAGE.

Recent studies reported structural, metabolic, and functional alterations in the frontal regions during the acute, subacute, and long-COVID phases (Anzalone et al., 2020; Duan et al., 2021; Guedj et al., 2021; Hosp et al., 2021; Kas et al., 2021). Unlike those studies, our work focused on hospitalized patients with neurological symptoms. Lower cortical GMV in the orbitofrontal cortex vs. controls was not an unexpected result in this patient group. This cortical area is a secondary olfactory cortex and part of a possible direct SARS-CoV-2 central nervous system invasion pathway (Baig and Sanders, 2020; Meinhardt et al., 2020; Bougakov et al., 2021).

Our results—especially the finding of a reduction in CTh and GMV in the orbitofrontal and cingulate cortex—confirm and extend very recent findings that were reported in a large cohort of SARS-CoV-2 infected patients obtained from the UK Biobank (Douaud et al., 2021): in fact, they demonstrated that the same regions that were altered in mildly infected patients are altered in patients with a severe form of the disease, pointing at a relationship between SARS-CoV-2 infection and gray matter alterations.

On the other hand, volumetric changes in the gray matter within the cingulate gyrus have also been described in post-COVID patients, which correlated to the loss of smell during the acute phase (Lu et al., 2020). In this respect, the vulnerability of the cingulate cortex to COVID-19 has been linked to its rich concentration of angiotensin-converting enzyme 2 (ACE-2), where the virus binds through its spike protein (Ibi et al., 2019; Ni et al., 2020). Interestingly, both regions, the orbitofrontal and cingulate cortex play essential roles in different cognitive functions such as attention, motivation, decision making, and conflict-error monitoring, which are impaired in COVID-19 patients (Ibi et al., 2019; Lu et al., 2020).

As to the possible mechanisms leading to lower GMV in these areas, this may be due to direct viral damage, especially to the olfactory cortex (Gori et al., 2020; Meinhardt et al., 2020). In addition, other possible causes might have contributed to these findings, such as a reduced oxygen supply to the brain (Solomon et al., 2020), post-infectious inflammation, cytokine-related hyperinflammation, and complications due to a coagulopathic state (Koralnik and Tyler, 2020; Pezzini and Padovani, 2020). Furthermore, without pre-COVID MRI studies, we cannot exclude that prior anatomical characteristics (i.e., atrophy) might be implicated in our results.

Another factor influencing our findings is the heterogeneity of the cohort’s pre-existing risk factors. In hospitalized SARS-CoV-2 infected patients, this is not unexpected. They were affected by several of the 24 demographic and health risk factors categories associated with severe disease outcomes (https://www.cdc.gov/coronavirus/2019-ncov/hcp/clinical-care/underlyingconditions.html, Updated June 15, 2022). Indeed, these risk factors may alter the brain’s gray matter volume and cortical thickness.

We considered the sample characteristics, including the pre-existing risk factors heterogeneity, as a strength of our study. The patient group we enrolled in this study represents the real-world characteristics of severely infected COVID-19 patients during the recent pandemic.

Favoring the hypothesis that a virus-triggered inflammatory process may represent the underlying cause of the observed cortical changes, we found a strong association between the GMV and CTh of frontal, orbitofrontal, temporal, parietal, and limbic cortices and an increase in the CSF/blood-albumin-ratio, CSF total protein levels, and CSF EN-RAGE levels. Our results are in line with a comprehensive study on the CSF profile in patients with COVID-19 and neurological symptoms (Jarius et al., 2022). The authors found a persistently elevated CSF/blood-albumin ratio, CSF total protein levels, CSF lactate levels, and inflammatory cytokines in COVID-19 patients.

An increase in the CSF/blood-albumin ratio points toward an impaired blood-brain barrier. This finding has been frequently reported in COVID-19 patients with neurological symptoms (Jarius et al., 2022). It has been related to several mechanisms such as a direct viral infection, non-specific inflammatory damage (Perico et al., 2020; Varga et al., 2020; Huang et al., 2021), a virus-induced anti-endothelial auto-immunity (Shi et al., 2022), and hypoxia-related alterations (Ackermann et al., 2020; Buja et al., 2020). However, an increase in the CSF/blood-albumin-ratio might also be caused by changes in the CSF production and resorption (Pellegrini, 2020; McMahon et al., 2021) or viral infection of the choroid plexus (Yang et al., 2021). Similarly, an increase in CSF total protein levels is often found in COVID-19 patients and interpreted as an indirect sign of an inflammatory response (Tandon et al., 2021).

Further, our study revealed that an increase in CSF EN-RAGE levels is associated with a widespread decrease in cortical GMV. EN-RAGE is a cytokine involved in an inflammatory cascade, leading to accelerated atherosclerosis (Ligthart et al., 2014). Increased CSF EN-RAGE levels may cause vascular alterations and consequently affect different organs, particularly in severe infections (Huang et al., 2020; Choudhary et al., 2021; Thepmankorn et al., 2021). Whether these factors contributed to the association of decreased GMV and CTh remains unknown. Future studies should investigate potential causal effects relating the production of EN-RAGE to cortical damage.

## Limitation

Our study has some limitations, encompassing the small sample size, the cross-sectional design, and the absence of pre-infection MRIs. Moreover, the lack of CSF and blood assessments in healthy subjects and some patients have undoubtedly limited the results’ interpretation and statistical power. Future studies should also be powered to consider the influence of comorbidities on brain alterations in neurologically affected SARS-CoV-2 infected patients. These comorbidities may modulate host-viral interactions, immunological and inflammatory responses, thereby contributing to different outcomes in COVID-19 patients.

In this study, we did not include a control group of patients with non-COVID-19 pneumonia, a condition that may lead to GMV alterations in several brain regions due to the decreased oxygenation to the cortex (Zhang et al., 2013; Harch and Fogarty, 2017). Future work should address these potential confounding factors and aim to elucidate whether the lack of adequate brain oxygenation rather than direct inflammatory/viral-triggered processes are responsible for neurodegenerative processes in this patient population. A voxel-wise/vertex-wise analysis will be implemented in future work to detect subtle morphological variations due to COVID-19.

Finally, follow-up data may allow exploring which cortical changes are associated with the acute disease or Post-COVID Syndrome. Whether the observed cortical alterations represent a consequence of viral infection is still to be determined. Future longitudinal studies should help elucidate the underlying causal mechanisms and clinical impact of these findings.

## Conclusion

In this study, using a multi-morphometric approach, we found that the gray matter volume in fronto-orbital and cingulate regions is affected in SARS-CoV-2 infected patients with neurological symptoms. The regional cortical GMV measure revealed brain changes in patients during the acute phase of COVID-19 infectious not shown by the cortical thickness and surface area measurements. The widespread cortical volumetric changes appear to be related to the infectious process and concomitant hyperinflammatory response represented by the CSF total protein values, CSF/blood-albumin ratio, and CSF EN-RAGE levels. Our results highlight the importance of considering cortical alteration patterns, particularly volumetric patterns, during the clinical assessment of neurologically affected COVID-19 patients. The study of this measure may improve clinical management and future cognitive rehabilitation programs.

## Data availability statement

The processed data supporting the conclusions of this article will be made available by the authors, without undue reservation. Requests to access the datasets should be directed to GS-D, gretel.sanabriadiaz@unibas.ch.

## Ethics statement

The studies involving human participants were reviewed and approved by the Ethics Committee of Northwestern and Central Switzerland (clinicaltrials.gov, NCT04472013, IRB approval EKNZ 2020-01503). The patients/participants provided their written informed consent to participate in this study.

## Author contributions

GS-D, ME, LM-G, and CG: study concepts and study design. JL, ME, M-NP, and GH: data acquisition. GS-D, ME, and LM-G: data analysis. GS-D, ME, LM-G, GH, and CG: data interpretation and manuscript editing. ME, JL, M-NP, and GH: clinical studies. GS-D and LM-G: statistical analysis. All authors: guarantors of the integrity of the study, manuscript drafting or manuscript revision for important intellectual content, approval of the final version of submitted manuscript, literature research, and agrees to ensure any questions related to the work are appropriately resolved.

## Footnotes

1
https://www.olink.com/products-services/target/neurology-panel/

2
https://www.olink.com/products-services/target/inflammation/

3
http://surfer.nmr.mgh.harvard.edu/

4
https://jasp-stats.org/

5
https://www.mathworks.com/

## Abbreviations

SARS-CoV-2: severe acute respiratory syndrome coronavirus 2
GMV: cortical gray matter volume
CTh: cortical thickness
SA: cortical surface area
FDR: false discovery rate
CSF: Cerebrospinal fluid
TIV: Total Intracranial Volume
TRANCE: tumor necrosis factor-related activation-induced cytokine
EN-RAGE: receptor for advanced glycation end-products binding protein
OPG: osteoprotegerin.
