# Supplementary material for: Brain cortical alterations in COVID-19 patients with neurological symptoms
Source: Front Neurosci. 2022 Oct 20;16:992165. doi: 10.3389/fnins.2022.992165 (PMC9630324; doi:10.3389/fnins.2022.992165)
Supplement: Supplementary file 1 [file Data_Sheet_1.docx]

Supplementary Material

**Supplementary Tables**

**Table 1**. CSF and Blood clinical parameters.

| **Lactate** | The CSF lactate concentration has been suggested as a valuable parameter to differentiate bacterial from viral meningitis (Huy et al., 2010). CSF lactate is produced by anaerobic metabolism, and the level increases in any condition, which causes a decrease in oxygen supply to the brain. There is no correlation with serum lactate level. |
| --- | --- |
| **CSF/blood albumin-ratio** | Albumin levels in the CSF and the serum are compared and represented in a ratio. It is helpful to measure the integrity of the blood-brain barrier (Jarius et al., 2022) . It can be affected in neuroinflammatory and cerebrovascular diseases; it is generally found to be a reliable sign of an active process, therefore, a potential marker of neuroinflammatory processes. |
| **Proteins** | CSF protein concentration may rise due to 2 factors: increased permeability of the blood-brain barrier allowing more protein and higher molecular weight proteins to enter the CSF or proteins may be synthesized within the cerebrospinal canal by inflammatory or other invading cells. Elevations in CSF total protein concentration can occur in infectious and non-infectious conditions (Kroksveen et al., 2011; Shahan et al., 2021). |
| **Leokocytes** | There are very few white blood cells present in CSF. A significant increase in white blood cells in the CSF can be caused by infection or inflammation of the central nervous system (Jarius et al., 2022).  Elevated WBC counts also may occur after a seizure,  in intracerebral hemorrhage, malignancy, and in a variety of inflammatory conditions (Drago et al., 2011; Moore & Barnett, 2016).  It may be indicative of meningitis, malignancy, or demyelinating disease. |
| **EN-RAGE** | EN-RAGE is a calcium-, zinc-, and copper-binding protein involved in the regulation of inflammatory processes and immune response (Meijer et al., 2012). It has been described to be involved in several inflammation-related diseases (Hudson & Lippman, 2018) (<https://www.olink.com/products-services/target/inflammation/biomarker/?biomarkerId=541>). |
| **OPG = TNFRSF11B** | OPG is a decoy receptor of cytokines TNFSF11 (RANKL) and possibly TNFSF10 (TRAIL) and belongs to the TNF receptor superfamily (*TNFRSF11B TNF Receptor Superfamily Member 11b [Homo Sapiens (Human)] - Gene - NCBI*, n.d.). OPG is up-regulated by estrogens and increasing calcium concentrations, and it has a role in transcriptional regulation in inflammation, innate immunity, and cell survival and differentiation (Bernardi et al., 2016). OPG has been described to be involved in several inflammation-related diseases (<https://www.olink.com/products-services/target/inflammation/biomarker/?biomarkerId=479>). Elements of the OPG pathway are involved in neuroinflammation and predominantly include glial cells. |
| **TRANCE** | TRANCE (TNFSF11, RANKL, OPGL) is a cytokine in the TNF family that, upon binding to its receptors RANK and OPG, plays important roles in dendritic cell maturation and survival, regulation of T cell-dependent immune responses, and stimulation of osteoclastogenesis (Josien et al., 1999). TRANCE has been described to be involved in several inflammation-related diseases (<https://www.olink.com/products-services/target/inflammation/biomarker/?biomarkerId=521>). TRANCE is known to counteract RANKL signaling, therefore displaying anti-inflammatory features. |

**Table 2.** List of pre-existing risk factors for the SARS-CoV-2-infected patient's group.

| **Sex** | **Age** | **Pre-existing risk factors** |
| --- | --- | --- |
| female | 66 | adipositas WHO grade III (BMI 42), arterial hypertension |
| female | 91 | metabolic syndrome, chronic kidney disease, atrial fibrillation |
| male | 79 | chronic lumbar backpain, atrial fibrillation |
| male | 29 | antibody deficiency disorder |
| female | 64 | chronic kidney disease, functional asplenia, steatosis hepatis |
| male | 70 | monoclonal gammopathy of unknown significance, lymphoplasmacytic lymphoma, diabetes mellitus, arterial hypertension |
| female | 64 | arterial hypertension |
| male | 70 | coronary heart disease |
| female | 47 | no pre-existing illness |
| male | 67 | cancer, diabetes mellitus, arterial hypertension |
| male | 68 | pulmonary hypertension, ventricular septal defect |
| female | 36 | no pre-existing illness |
| female | 82 | chronic obstructive pulmonary disease (COPD), arterial hypertension, chronic kidney disease |
| female | 50 | headache |
| female | 38 | no pre-existing illness |
| male | 24 | asthma |
| male | 44 | no pre-existing illness |
| female | 36 | myasthenia gravis |
| female | 27 | no pre-existing illness |
| male | 75 | coronary heart disease |
| female | 22 | no pre-existing illness |
| male | 63 | coronary heart disease, dilatative cardiomyopathy |
| female | 58 | no pre-existing illness |
| female | 38 | no pre-existing illness |
| female | 60 | chronic lymphatic leukaemia (CLL) |
| female | 38 | hypothyroidism |
| male | 57 | acute myeloid leukaemia (AML), steroid-induced diabetes mellitus |
| female | 24 | no pre-existing illness |
| male | 49 | no pre-existing illness |
| female | 28 | no pre-existing illness |
| male | 56 | no pre-existing illness |
| male | 59 | hypercholesterolemia |
| female | 24 | no pre-existing illness |

**Table 3.** Neurological symptoms required an MRI study in the SARS-CoV-2-infected patients.

| **Sex** | **Age** | **Neurologic symptoms/ reasons for MRI study** |
| --- | --- | --- |
| female | 66 | prolonged reduced vigilance in the intensive care unit |
| female | 91 | a short episode of aphasia |
| male | 79 | seizures |
| male | 29 | confusion, dizziness |
| female | 64 | prolonged reduced vigilance in the intensive care unit |
| male | 70 | delirious symptoms and confusion |
| female | 64 | headache, loss of smell and dysgeusia |
| male | 70 | headache, loss of smell and dysgeusia |
| female | 47 | (inflammatory) polyneuropathy |
| male | 67 | Dizziness, loss of smell and dysgeusia |
| male | 68 | loss of smell |
| female | 36 | dizziness, loss of smell and dysgeusia |
| female | 82 | confusion, reduced vigilance |
| female | 50 | headache with nausea and vomiting |
| female | 38 | generalized tonic-clonic seizure |
| male | 24 | headache, loss of smell and dysgeusia |
| male | 44 | vertigo and nystagmus |
| female | 36 | loss of smell |
| female | 27 | loss of smell, fatigue |
| male | 75 | confusion, dizziness |
| female | 22 | loss of smell, headache with nausea |
| male | 63 | headache, cephalgia |
| female | 58 | generalized tonic-clonic seizure |
| female | 38 | headache, confusion, loss of smell |
| female | 60 | loss of smell, dysgeusia, dizziness |
| female | 38 | headache, cephalgia |
| male | 57 | aphasia, mutism |
| female | 24 | headache, loss of smell |
| male | 49 | delirious symptoms and reduced vigilance |
| female | 28 | headache |
| male | 56 | delirious symptoms |
| male | 59 | headache |
| female | 24 | headache, loss of smell, fatigue |

**Table 4.** Results of the cortical thickness contrast between patients and control (linear regression analysis)

| **Structures** | **Control mean** | **Control std** | **Patient mean** | **Patient std** | **t stat** | **p-val uncor** | **p-val corr** |
| --- | --- | --- | --- | --- | --- | --- | --- |
| R caudal anterior cingulate | 2.47 | 0.22 | 2.39 | 0.18 | 0.25 | 0.38 | 0.40 |
| R caudal middle frontal | 2.44 | 0.23 | 2.38 | 0.25 | 1.00 | 0.24 | 0.40 |
| R cuneus | 1.92 | 0.17 | 1.86 | 0.20 | 0.50 | 0.35 | 0.40 |
| R enthorinal | 3.38 | 0.39 | 3.39 | 0.38 | -0.62 | 0.33 | 0.40 |
| R fusiform | 2.71 | 0.17 | 2.60 | 0.20 | 1.24 | 0.18 | 0.40 |
| R inferior parietal | 2.39 | 0.20 | 2.35 | 0.16 | -0.53 | 0.34 | 0.40 |
| R inferior temporal | 2.73 | 0.20 | 2.62 | 0.18 | 0.43 | 0.36 | 0.40 |
| R isthmus cingulate | 2.30 | 0.21 | 2.30 | 0.22 | -1.29 | 0.17 | 0.40 |
| R lateral occipital | 2.21 | 0.17 | 2.15 | 0.14 | 1.18 | 0.20 | 0.40 |
| R lateral orbitof rontal | 2.56 | 0.16 | 2.52 | 0.19 | 0.86 | 0.27 | 0.40 |
| R lingual | 2.04 | 0.12 | 1.99 | 0.15 | 0.87 | 0.27 | 0.40 |
| R medial orbito frontal | 2.41 | 0.18 | 2.35 | 0.18 | 0.45 | 0.36 | 0.40 |
| R middle temporal | 2.72 | 0.20 | 2.59 | 0.21 | 0.45 | 0.36 | 0.40 |
| R parahippocampal | 2.68 | 0.28 | 2.63 | 0.25 | 0.06 | 0.40 | 0.40 |
| R paracentral | 2.39 | 0.20 | 2.32 | 0.23 | 0.47 | 0.35 | 0.40 |
| R pars opercularis | 2.46 | 0.19 | 2.42 | 0.21 | -1.27 | 0.18 | 0.40 |
| R pars orbitalis | 2.58 | 0.23 | 2.63 | 0.21 | -1.19 | 0.20 | 0.40 |
| R pars triangularis | 2.34 | 0.23 | 2.31 | 0.22 | 0.24 | 0.39 | 0.40 |
| R pericalcarine | 1.58 | 0.11 | 1.59 | 0.31 | 0.07 | 0.40 | 0.40 |
| R postcentral | 1.99 | 0.16 | 1.93 | 0.19 | 1.37 | 0.15 | 0.40 |
| R posterior cingulate | 2.39 | 0.19 | 2.35 | 0.24 | -0.27 | 0.38 | 0.40 |
| R precentral | 2.47 | 0.20 | 2.37 | 0.29 | 0.33 | 0.38 | 0.40 |
| R precuneus | 2.32 | 0.21 | 2.28 | 0.17 | -0.89 | 0.27 | 0.40 |
| R rostral anterior cingulate | 2.86 | 0.27 | 2.73 | 0.23 | 0.48 | 0.35 | 0.40 |
| R rostral middle frontal | 2.24 | 0.19 | 2.23 | 0.19 | 0.33 | 0.38 | 0.40 |
| R superior frontal | 2.55 | 0.24 | 2.59 | 0.23 | -1.98 | 0.06 | 0.40 |
| R superior parietal | 2.12 | 0.19 | 2.09 | 0.16 | -0.51 | 0.35 | 0.40 |
| R superior temporal | 2.69 | 0.23 | 2.56 | 0.22 | 0.92 | 0.26 | 0.40 |
| R supramarginal | 2.43 | 0.20 | 2.34 | 0.19 | -0.41 | 0.37 | 0.40 |
| R frontal pole | 2.61 | 0.28 | 2.54 | 0.30 | -0.21 | 0.39 | 0.40 |
| R temporal pole | 3.57 | 0.36 | 3.50 | 0.38 | 0.66 | 0.32 | 0.40 |
| R transverse temporal | 2.31 | 0.29 | 2.28 | 0.34 | -1.82 | 0.08 | 0.40 |
| R insula | 2.92 | 0.23 | 2.80 | 0.26 | 1.20 | 0.19 | 0.40 |
| L caudal anterior cingulate | 2.61 | 0.29 | 2.61 | 0.25 | -1.14 | 0.21 | 0.40 |
| L caudal middle frontal | 2.46 | 0.25 | 2.42 | 0.22 | -0.45 | 0.36 | 0.40 |
| L cuneus | 1.84 | 0.15 | 1.80 | 0.23 | 0.28 | 0.38 | 0.40 |
| L enthorinal | 3.28 | 0.28 | 3.31 | 0.33 | -1.28 | 0.17 | 0.40 |
| L fusiform | 2.68 | 0.17 | 2.60 | 0.23 | 0.30 | 0.38 | 0.40 |
| L inferior parietal | 2.34 | 0.20 | 2.33 | 0.21 | -1.33 | 0.16 | 0.40 |
| L inferior temporal | 2.71 | 0.18 | 2.64 | 0.19 | -0.42 | 0.36 | 0.40 |
| L isthmus cingulate | 2.30 | 0.24 | 2.24 | 0.24 | -0.38 | 0.37 | 0.40 |
| L lateral occipital | 2.13 | 0.16 | 2.09 | 0.16 | -0.14 | 0.39 | 0.40 |
| L lateral orbitofrontal | 2.59 | 0.17 | 2.53 | 0.22 | 0.21 | 0.39 | 0.40 |
| L lingual | 2.02 | 0.16 | 1.93 | 0.19 | 0.49 | 0.35 | 0.40 |
| L medial orbitofrontal | 2.40 | 0.18 | 2.37 | 0.20 | 0.37 | 0.37 | 0.40 |
| L middle temporal | 2.69 | 0.20 | 2.63 | 0.20 | -0.98 | 0.25 | 0.40 |
| L parahippocampal | 2.70 | 0.45 | 2.75 | 0.29 | -1.10 | 0.22 | 0.40 |
| L paracentral | 2.35 | 0.18 | 2.27 | 0.25 | -0.76 | 0.30 | 0.40 |
| L pars opercularis | 2.48 | 0.21 | 2.41 | 0.22 | -0.25 | 0.39 | 0.40 |
| L pars orbitalis | 2.63 | 0.22 | 2.57 | 0.23 | 0.89 | 0.27 | 0.40 |
| L pars triangularis | 2.39 | 0.23 | 2.32 | 0.27 | 0.32 | 0.38 | 0.40 |
| L pericalcarine | 1.56 | 0.11 | 1.57 | 0.22 | -0.21 | 0.39 | 0.40 |
| L postcentral | 1.99 | 0.17 | 1.96 | 0.18 | 0.48 | 0.35 | 0.40 |
| L posterior cingulate | 2.45 | 0.20 | 2.35 | 0.23 | 0.23 | 0.39 | 0.40 |
| L precentral | 2.53 | 0.20 | 2.40 | 0.25 | 1.59 | 0.11 | 0.40 |
| L precuneus | 2.29 | 0.18 | 2.24 | 0.21 | -1.03 | 0.23 | 0.40 |
| L rostral anterior cingulate | 2.75 | 0.26 | 2.73 | 0.26 | -0.92 | 0.26 | 0.40 |
| L rostral middle frontal | 2.29 | 0.20 | 2.29 | 0.18 | -0.29 | 0.38 | 0.40 |
| L superior frontal | 2.59 | 0.27 | 2.60 | 0.25 | -1.55 | 0.12 | 0.40 |
| L superior parietal | 2.13 | 0.19 | 2.10 | 0.17 | -0.71 | 0.31 | 0.40 |
| L superior rtemporal | 2.69 | 0.24 | 2.53 | 0.23 | 1.70 | 0.09 | 0.40 |
| L supramarginal | 2.40 | 0.22 | 2.35 | 0.20 | -1.74 | 0.09 | 0.40 |
| L frontal pole | 2.68 | 0.25 | 2.62 | 0.28 | 0.43 | 0.36 | 0.40 |
| L temporal pole | 3.49 | 0.32 | 3.32 | 0.43 | 1.19 | 0.19 | 0.40 |
| L transverse temporal | 2.34 | 0.24 | 2.21 | 0.32 | 0.60 | 0.33 | 0.40 |
| L insula | 2.94 | 0.23 | 2.83 | 0.28 | 1.06 | 0.23 | 0.40 |

*R: right, L:left, std:standard deviation, p-val uncor: p-values uncorrected, p-val corr: p-values corrected by FDR.*

**Table 5.** Results of the gray matter volume contrast between patients and control (linear regression analysis)

| **Structures** | **Control mean** | **Control std** | **Patient mean** | **Patient std** | **T stat** | **P-val uncor** | **P-val corr** |
| --- | --- | --- | --- | --- | --- | --- | --- |
| R caudal anterior cingulate | 1.98 | 0.46 | 1.69 | 0.31 | 1.85 | 0.07 | 0.21 |
| R caudal middle frontal | 5.81 | 1.29 | 5.01 | 1.29 | 2.67 | **0.01** | 0.08 |
| R cuneus | 3.27 | 0.54 | 3.02 | 0.57 | 1.59 | 0.11 | 0.24 |
| R enthorinal | 1.85 | 0.43 | 2.04 | 0.50 | -1.72 | 0.09 | 0.23 |
| R fusiform | 8.95 | 1.53 | 8.18 | 1.72 | 1.76 | 0.09 | 0.23 |
| R inferior parietal | 13.26 | 2.49 | 12.69 | 2.74 | -0.10 | 0.40 | 0.40 |
| R inferior temporal | 10.13 | 2.10 | 9.00 | 2.09 | 1.46 | 0.14 | 0.24 |
| R isthmus cingulate | 2.33 | 0.43 | 2.35 | 0.47 | -0.63 | 0.32 | 0.40 |
| R lateral occipital | 11.69 | 2.14 | 10.76 | 2.05 | 2.07 | 0.05 | 0.19 |
| R lateral orbitofrontal | 7.16 | 0.99 | 6.83 | 1.31 | 1.67 | 0.10 | 0.23 |
| R lingual | 6.99 | 0.80 | 6.33 | 1.29 | 1.68 | 0.10 | 0.23 |
| R medial orbitofrontal | 5.19 | 0.74 | 4.96 | 0.91 | 0.14 | 0.39 | 0.40 |
| R middle temporal | 10.90 | 1.86 | 9.73 | 1.80 | 1.97 | 0.06 | 0.19 |
| R parahippocampal | 1.97 | 0.37 | 1.89 | 0.34 | 0.04 | 0.40 | 0.40 |
| R paracentral | 3.99 | 0.63 | 3.67 | 0.67 | 1.88 | 0.07 | 0.21 |
| R pars opercularis | 3.61 | 0.61 | 3.22 | 0.61 | 2.67 | **0.01** | 0.08 |
| R pars orbitalis | 2.59 | 0.53 | 2.54 | 0.45 | 1.05 | 0.23 | 0.32 |
| R pars triangularis | 3.98 | 0.83 | 3.63 | 0.78 | 2.71 | **0.01** | 0.08 |
| R pericalcarine | 2.22 | 0.34 | 2.07 | 0.46 | 1.48 | 0.13 | 0.24 |
| R postcentral | 8.75 | 1.56 | 7.93 | 1.43 | 2.36 | **0.03** | 0.15 |
| R posterior cingulate | 3.04 | 0.57 | 2.80 | 0.52 | 1.49 | 0.13 | 0.24 |
| R precentral | 12.80 | 1.82 | 11.86 | 2.15 | 2.14 | **0.04** | 0.18 |
| R precuneus | 9.27 | 1.59 | 8.92 | 1.83 | 0.35 | 0.37 | 0.40 |
| R rostral anterior cingulate | **1.90** | **0.38** | **1.64** | **0.36** | **3.68** | **0.00** | **0.04** |
| R rostral middle frontal | 14.60 | 2.54 | 13.79 | 2.88 | 2.08 | 0.05 | 0.19 |
| R superior frontal | 20.72 | 3.90 | 19.21 | 3.77 | 2.75 | **0.01** | 0.08 |
| R superior parietal | 11.77 | 1.74 | 11.39 | 2.06 | 0.56 | 0.34 | 0.40 |
| R superior temporal | 11.02 | 1.53 | 10.03 | 2.04 | 1.35 | 0.16 | 0.25 |
| R supramarginal | 9.21 | 1.50 | 8.43 | 1.52 | 1.37 | 0.16 | 0.25 |
| R frontal pole | 1.09 | 0.17 | 0.97 | 0.16 | 1.70 | 0.09 | 0.23 |
| R temporal pole | 2.47 | 0.45 | 2.41 | 0.69 | 0.60 | 0.33 | 0.40 |
| R transverse temporal | 0.85 | 0.14 | 0.77 | 0.18 | 1.47 | 0.13 | 0.24 |
| R insula | 7.13 | 1.01 | 6.32 | 0.96 | 2.75 | **0.01** | 0.08 |
| L caudal anterior cingulate | 1.66 | 0.53 | 1.62 | 0.47 | -0.21 | 0.39 | 0.40 |
| L caudal middle frontal | 6.20 | 1.21 | 5.39 | 1.32 | 2.67 | **0.01** | 0.08 |
| L cuneus | 2.85 | 0.42 | 2.69 | 0.58 | 0.33 | 0.38 | 0.40 |
| L enthorinal | 1.94 | 0.33 | 2.04 | 0.45 | -1.22 | 0.19 | 0.28 |
| L fusiform | 9.19 | 1.58 | 8.60 | 1.51 | 2.02 | 0.05 | 0.19 |
| L inferior parietal | 10.48 | 2.02 | 10.30 | 1.99 | -0.31 | 0.38 | 0.40 |
| L inferior temporal | 10.34 | 1.79 | 9.57 | 1.87 | 0.92 | 0.26 | 0.35 |
| L isthmus cingulate | 2.49 | 0.44 | 2.45 | 0.46 | -0.78 | 0.29 | 0.38 |
| L lateral occipital | 11.30 | 1.97 | 10.59 | 1.85 | 1.09 | 0.22 | 0.31 |
| L lateral orbito frontal | 7.42 | 1.04 | 7.05 | 1.27 | 1.55 | 0.12 | 0.24 |
| L lingual | 6.41 | 0.87 | 5.97 | 1.22 | 0.56 | 0.34 | 0.40 |
| L medial orbito frontal | **5.08** | **0.84** | **4.71** | **0.72** | **3.35** | **0.00** | **0.04** |
| L middle temporal | 9.99 | 2.02 | 9.25 | 1.79 | 1.29 | 0.17 | 0.27 |
| L parahippocampal | 2.03 | 0.40 | 2.04 | 0.34 | -0.48 | 0.35 | 0.40 |
| L paracentral | 3.52 | 0.44 | 3.26 | 0.68 | 1.45 | 0.14 | 0.24 |
| L pars opercularis | 4.36 | 0.88 | 4.09 | 1.01 | 1.71 | 0.09 | 0.23 |
| L pars orbitalis | 2.28 | 0.40 | 2.14 | 0.37 | 1.58 | 0.11 | 0.24 |
| L pars triangularis | 3.49 | 0.77 | 3.31 | 0.76 | 0.99 | 0.24 | 0.33 |
| L pericalcarine | 1.90 | 0.36 | 1.87 | 0.45 | 0.25 | 0.38 | 0.40 |
| L postcentral | 8.94 | 1.27 | 8.37 | 1.32 | 1.99 | 0.06 | 0.19 |
| L posterior cingulate | 2.99 | 0.60 | 2.82 | 0.60 | 0.75 | 0.30 | 0.38 |
| L precentral | 13.17 | 1.74 | 11.88 | 2.09 | 2.90 | **0.01** | 0.08 |
| L precuneus | 8.90 | 1.52 | 8.69 | 1.71 | -0.03 | 0.40 | 0.40 |
| L rostral anterior cingulate | 2.60 | 0.53 | 2.17 | 0.58 | 2.30 | **0.03** | 0.15 |
| L rostral middle frontal | 14.18 | 2.45 | 13.38 | 2.57 | 2.19 | **0.04** | 0.18 |
| L superiorfrontal | **22.00** | **3.75** | **19.87** | **3.47** | **3.35** | **0.00** | **0.04** |
| L superior parietal | 12.43 | 2.17 | 11.58 | 1.94 | 1.41 | 0.15 | 0.24 |
| L superior temporal | 11.88 | 1.88 | 10.70 | 1.77 | 2.80 | **0.01** | 0.08 |
| L supramarginal | 10.37 | 1.67 | 9.52 | 1.79 | 1.64 | 0.10 | 0.23 |
| L frontal pole | 0.92 | 0.17 | 0.87 | 0.16 | 0.82 | 0.28 | 0.37 |
| L temporal pole | 2.38 | 0.36 | 2.25 | 0.48 | 1.14 | 0.21 | 0.30 |
| L transverse temporal | 1.13 | 0.22 | 1.05 | 0.31 | 1.95 | 0.06 | 0.19 |
| L insula | 7.08 | 0.78 | 6.40 | 1.14 | 1.47 | 0.13 | 0.24 |

*R: right, L:left, std:standard deviation, p-val uncor: p-values uncorrected, p-val corr: p-values corrected by FDR. In bold corrected and uncorrected p-values <0.05.*

**Table 6.** Results of the surface area contrast between patients and control (linear regression analysis)

| **Structures** | **Control mean** | **Control std** | **Patient mean** | **Patient std** | **t stat** | **p-val uncor** | **p-val corr** |
| --- | --- | --- | --- | --- | --- | --- | --- |
| R caudal anterior cingulate | 7.15 | 1.37 | 6.41 | 0.97 | 1.16 | 0.20 | 0.40 |
| R caudal middle frontal | 21.45 | 3.47 | 19.37 | 3.83 | 1.15 | 0.20 | 0.40 |
| R cuneus | 15.64 | 1.92 | 15.14 | 2.74 | -0.55 | 0.34 | 0.40 |
| R enthorinal | 3.80 | 0.84 | 4.02 | 0.81 | -0.92 | 0.26 | 0.40 |
| R fusiform | 28.89 | 4.06 | 27.39 | 4.85 | -0.19 | 0.39 | 0.40 |
| R inferior parietal | 50.30 | 7.44 | 48.81 | 9.41 | -1.69 | 0.10 | 0.40 |
| R inferior temporal | 31.30 | 5.32 | 28.90 | 5.73 | 0.12 | 0.39 | 0.40 |
| R isthmus cingulate | 8.99 | 1.04 | 9.13 | 1.55 | -1.14 | 0.21 | 0.40 |
| R lateral occipital | 47.92 | 6.43 | 46.23 | 8.87 | -0.06 | 0.40 | 0.40 |
| R lateral orbitofrontal | 25.58 | 3.16 | 25.01 | 3.80 | -0.30 | 0.38 | 0.40 |
| R lingual | 31.93 | 3.50 | 29.96 | 6.21 | 0.11 | 0.40 | 0.40 |
| R medial orbitofrontal | 19.08 | 2.31 | 18.52 | 2.35 | -0.64 | 0.32 | 0.40 |
| R middle temporal | 33.27 | 4.11 | 30.83 | 4.99 | 0.56 | 0.34 | 0.40 |
| R parahippocampal | 6.27 | 0.85 | 6.19 | 1.10 | 0.11 | 0.39 | 0.40 |
| R paracentral | 15.57 | 1.92 | 14.98 | 2.06 | 0.64 | 0.32 | 0.40 |
| R pars opercularis | 13.18 | 1.80 | 12.08 | 2.00 | 2.07 | 0.05 | 0.40 |
| R pars orbitalis | 7.96 | 1.23 | 7.71 | 1.10 | 0.05 | 0.40 | 0.40 |
| R parstr iangularis | 15.02 | 2.22 | 14.13 | 2.61 | 0.86 | 0.27 | 0.40 |
| R pericalcarine | 15.73 | 2.10 | 14.77 | 3.04 | -0.08 | 0.40 | 0.40 |
| R postcentral | 39.75 | 5.34 | 37.52 | 4.95 | 0.27 | 0.38 | 0.40 |
| R posterior cingulate | 11.54 | 1.75 | 11.15 | 1.84 | 0.25 | 0.38 | 0.40 |
| R precentral | 48.22 | 5.08 | 47.16 | 7.26 | 0.08 | 0.40 | 0.40 |
| R precuneus | 37.94 | 5.26 | 37.05 | 6.58 | -1.01 | 0.24 | 0.40 |
| R rostral anterior cingulate | 5.81 | 1.22 | 5.40 | 0.94 | 2.00 | 0.06 | 0.40 |
| R rostral middle frontal | 57.06 | 8.27 | 55.62 | 10.07 | -0.20 | 0.39 | 0.40 |
| R superior frontal | 70.36 | 9.72 | 65.74 | 11.00 | 1.79 | 0.08 | 0.40 |
| R superior parietal | 50.75 | 6.63 | 49.80 | 7.11 | -1.19 | 0.19 | 0.40 |
| R superior temporal | 36.09 | 3.62 | 34.23 | 5.83 | 0.17 | 0.39 | 0.40 |
| R supramarginal | 34.81 | 4.96 | 32.75 | 4.73 | 0.29 | 0.38 | 0.40 |
| R frontal pole | 3.00 | 0.34 | 2.95 | 0.43 | -1.24 | 0.18 | 0.40 |
| R temporal pole | 4.69 | 0.64 | 4.73 | 1.50 | 0.08 | 0.40 | 0.40 |
| R transverse temporal | 3.22 | 0.39 | 3.03 | 0.58 | 0.56 | 0.34 | 0.40 |
| R insula | 23.93 | 3.47 | 22.51 | 2.81 | 0.73 | 0.30 | 0.40 |
| L caudal anterior cingulate | 6.09 | 1.51 | 5.94 | 1.26 | -0.69 | 0.31 | 0.40 |
| L caudal middle frontal | 23.12 | 3.41 | 20.66 | 4.67 | 1.33 | 0.16 | 0.40 |
| L cuneus | 14.70 | 1.67 | 14.30 | 2.67 | -1.42 | 0.15 | 0.40 |
| L enthorinal | 4.26 | 0.82 | 4.49 | 0.98 | -0.99 | 0.24 | 0.40 |
| L fusiform | 29.71 | 4.01 | 28.91 | 4.43 | 0.42 | 0.36 | 0.40 |
| L inferior parietal | 40.80 | 5.80 | 40.75 | 7.23 | -2.33 | **0.03** | 0.40 |
| L inferior temporal | 31.91 | 4.74 | 30.30 | 6.17 | -0.50 | 0.35 | 0.40 |
| L isthmus cingulate | 9.91 | 1.31 | 10.21 | 1.57 | -2.41 | **0.02** | 0.40 |
| L lateral occipital | 48.54 | 5.84 | 47.03 | 7.57 | -0.23 | 0.39 | 0.40 |
| L lateral orbitofrontal | 26.34 | 3.15 | 25.66 | 3.57 | -0.56 | 0.34 | 0.40 |
| L lingual | 29.85 | 3.39 | 29.18 | 4.89 | -1.02 | 0.23 | 0.40 |
| L medial orbitofrontal | 19.05 | 2.65 | 17.95 | 2.14 | 2.09 | 0.05 | 0.40 |
| L middle temporal | 30.02 | 4.39 | 28.36 | 5.04 | -0.13 | 0.39 | 0.40 |
| L parahippocampal | 6.45 | 0.85 | 6.35 | 0.63 | 0.24 | 0.39 | 0.40 |
| L paracentral | 13.88 | 1.59 | 13.58 | 2.45 | 0.63 | 0.33 | 0.40 |
| L pars opercularis | 15.66 | 2.19 | 15.05 | 2.93 | 1.14 | 0.21 | 0.40 |
| L pars orbitalis | 6.74 | 0.95 | 6.54 | 1.06 | -0.62 | 0.33 | 0.40 |
| L pars triangularis | 12.92 | 1.79 | 12.71 | 2.29 | -0.27 | 0.38 | 0.40 |
| L pericalcarine | 13.71 | 2.05 | 13.45 | 2.49 | -0.89 | 0.27 | 0.40 |
| L postcentral | 40.61 | 4.37 | 38.42 | 4.62 | 0.13 | 0.39 | 0.40 |
| L posterior cingulate | 11.29 | 1.56 | 11.29 | 1.86 | -0.86 | 0.27 | 0.40 |
| L precentral | 48.31 | 5.23 | 46.01 | 7.48 | 0.41 | 0.36 | 0.40 |
| L precuneus | 36.65 | 5.18 | 36.86 | 6.16 | -2.24 | **0.03** | 0.40 |
| L rostral anterior cingulate | 8.39 | 1.68 | 7.18 | 1.82 | 1.69 | 0.10 | 0.40 |
| L rostral middle frontal | 54.82 | 7.16 | 52.42 | 9.67 | -0.11 | 0.39 | 0.40 |
| L superior frontal | 74.13 | 10.01 | 68.33 | 11.35 | 1.54 | 0.12 | 0.40 |
| L superior parietal | 53.27 | 7.48 | 50.61 | 7.84 | 0.32 | 0.38 | 0.40 |
| L superior temporal | 38.46 | 4.77 | 36.81 | 5.28 | 0.69 | 0.31 | 0.40 |
| L supramarginal | 39.27 | 5.78 | 36.57 | 6.42 | 0.78 | 0.29 | 0.40 |
| L frontal pole | 2.48 | 0.32 | 2.37 | 0.31 | 0.70 | 0.31 | 0.40 |
| L temporal pole | 4.57 | 0.69 | 4.66 | 0.64 | -1.10 | 0.22 | 0.40 |
| L transverse temporal | 4.41 | 0.68 | 4.34 | 0.94 | 0.91 | 0.26 | 0.40 |
| L insula | 24.11 | 2.91 | 22.84 | 3.29 | -0.52 | 0.35 | 0.40 |

*R: right, L:left, std:standard deviation, p-val uncor: p-values uncorrected, p-val corr: p-values corrected by FDR. In bold corrected and uncorrected p-values <0.05.*

**Table 7.** Results of the gray matter volume contrast between Class I and Class II-III for a subgroup of patients with CSF/Blood tests (linear regression analysis)

| **Structures** | **Control mean** | **Control std** | **Patient mean** | **Patient std** | **t stat** | **p- val uncor** | **p-val corr** |
| --- | --- | --- | --- | --- | --- | --- | --- |
| R caudal anterior cingulate | 1.69 | 0.31 | 1.61 | 0.35 | 2.30 | **0.03** | 0.20 |
| R caudal middle frontal | 5.01 | 1.29 | 4.90 | 1.29 | 0.76 | 0.29 | 0.40 |
| R cuneus | 3.02 | 0.57 | 3.06 | 0.62 | 0.00 | 0.40 | 0.40 |
| R enthorinal | 2.04 | 0.50 | 2.05 | 0.47 | 0.39 | 0.36 | 0.40 |
| R fusiform | 8.18 | 1.72 | 7.89 | 1.67 | 0.83 | 0.28 | 0.40 |
| R inferior parietal | 12.69 | 2.74 | 12.12 | 2.90 | 1.92 | 0.07 | 0.23 |
| R inferior temporal | 9.00 | 2.09 | 8.72 | 2.25 | 0.66 | 0.31 | 0.40 |
| R isthmus cingulate | 2.35 | 0.47 | 2.33 | 0.46 | 0.64 | 0.32 | 0.40 |
| R lateral occipital | 10.76 | 2.05 | 10.59 | 1.98 | 0.40 | 0.36 | 0.40 |
| R lateral orbitofrontal | 6.83 | 1.31 | 6.58 | 1.47 | 1.30 | 0.17 | 0.40 |
| R lingual | 6.33 | 1.29 | 6.12 | 0.98 | 0.49 | 0.35 | 0.40 |
| R medial orbitofrontal | 4.96 | 0.91 | 4.89 | 0.96 | 1.18 | 0.19 | 0.40 |
| R middle temporal | 9.73 | 1.80 | 9.71 | 2.25 | 0.57 | 0.33 | 0.40 |
| R parahippocampal | 1.89 | 0.34 | 1.83 | 0.27 | 0.45 | 0.36 | 0.40 |
| R paracentral | 3.67 | 0.67 | 3.46 | 0.71 | 1.41 | 0.15 | 0.39 |
| R pars opercularis | 3.22 | 0.61 | 3.11 | 0.51 | 0.29 | 0.38 | 0.40 |
| R pars orbitalis | 2.54 | 0.45 | 2.43 | 0.42 | 1.09 | 0.22 | 0.40 |
| R pars triangularis | 3.63 | 0.78 | 3.47 | 0.49 | 0.73 | 0.30 | 0.40 |
| R pericalcarine | 2.07 | 0.46 | 2.02 | 0.38 | 1.06 | 0.22 | 0.40 |
| R postcentral | 7.93 | 1.43 | 7.48 | 1.03 | 1.65 | 0.10 | 0.31 |
| R posterior cingulate | 2.80 | 0.52 | 2.66 | 0.45 | 3.49 | **0.00** | 0.15 |
| R precentral | 11.86 | 2.15 | 11.19 | 2.13 | 2.16 | **0.04** | 0.20 |
| R precuneus | 8.92 | 1.83 | 8.88 | 1.62 | 0.35 | 0.37 | 0.40 |
| R rostral anterior cingulate | 1.64 | 0.36 | 1.52 | 0.34 | 2.23 | **0.04** | 0.20 |
| R rostral middle frontal | 13.79 | 2.88 | 13.62 | 3.01 | 0.06 | 0.39 | 0.40 |
| R superior frontal | 19.21 | 3.77 | 18.51 | 3.92 | 0.79 | 0.29 | 0.40 |
| R superior parietal | 11.39 | 2.06 | 11.23 | 2.45 | 1.21 | 0.19 | 0.40 |
| R superior temporal | 10.03 | 2.04 | 9.49 | 1.72 | 1.88 | 0.07 | 0.23 |
| R supramarginal | 8.43 | 1.52 | 8.28 | 1.70 | 1.79 | 0.08 | 0.26 |
| R frontal pole | 0.97 | 0.16 | 0.99 | 0.18 | -0.19 | 0.39 | 0.40 |
| R temporal pole | 2.41 | 0.69 | 2.29 | 0.40 | 0.55 | 0.34 | 0.40 |
| R transverse temporal | 0.77 | 0.18 | 0.72 | 0.19 | 3.03 | **0.01** | 0.16 |
| R insula | 6.32 | 0.96 | 6.18 | 0.90 | 1.36 | 0.16 | 0.40 |
| L caudal anterior cingulate | 1.62 | 0.47 | 1.58 | 0.56 | 0.11 | 0.39 | 0.40 |
| L caudal middle frontal | 5.39 | 1.32 | 5.14 | 1.42 | 0.73 | 0.30 | 0.40 |
| L cuneus | 2.69 | 0.58 | 2.65 | 0.64 | 0.93 | 0.25 | 0.40 |
| L enthorinal | 2.04 | 0.45 | 2.08 | 0.46 | -0.31 | 0.38 | 0.40 |
| L fusiform | 8.60 | 1.51 | 8.12 | 1.44 | 1.96 | 0.06 | 0.23 |
| L inferior parietal | 10.30 | 1.99 | 9.99 | 2.08 | 3.00 | **0.01** | 0.16 |
| L inferior temporal | 9.57 | 1.87 | 9.24 | 2.42 | 1.55 | 0.12 | 0.35 |
| L isthmus cingulate | 2.45 | 0.46 | 2.38 | 0.54 | 1.42 | 0.14 | 0.39 |
| L lateral occipital | 10.59 | 1.85 | 10.19 | 1.93 | 2.00 | 0.06 | 0.23 |
| L lateral orbitofrontal | 7.05 | 1.27 | 6.85 | 1.38 | 2.03 | 0.05 | 0.23 |
| L lingual | 5.97 | 1.22 | 6.05 | 1.20 | -1.30 | 0.17 | 0.40 |
| L medial orbitofrontal | 4.71 | 0.72 | 4.53 | 0.69 | 1.93 | 0.06 | 0.23 |
| L middle temporal | 9.25 | 1.79 | 8.79 | 2.13 | 2.59 | **0.02** | 0.20 |
| L parahippocampal | 2.04 | 0.34 | 1.96 | 0.29 | 1.00 | 0.24 | 0.40 |
| L paracentral | 3.26 | 0.68 | 3.29 | 0.81 | -0.81 | 0.28 | 0.40 |
| L pars opercularis | 4.09 | 1.01 | 3.97 | 1.21 | 0.36 | 0.37 | 0.40 |
| L pars orbitalis | 2.14 | 0.37 | 2.00 | 0.36 | 2.51 | **0.02** | 0.20 |
| L pars triangularis | 3.31 | 0.76 | 3.19 | 0.76 | 0.89 | 0.26 | 0.40 |
| L pericalcarine | 1.87 | 0.45 | 1.88 | 0.49 | -0.20 | 0.39 | 0.40 |
| L postcentral | 8.37 | 1.32 | 8.16 | 1.49 | 0.38 | 0.37 | 0.40 |
| L posterior cingulate | 2.82 | 0.60 | 2.67 | 0.58 | 2.21 | **0.04** | 0.20 |
| L precentral | 11.88 | 2.09 | 11.51 | 2.11 | 1.12 | 0.21 | 0.40 |
| L precuneus | 8.69 | 1.71 | 8.43 | 1.85 | 2.22 | **0.04** | 0.20 |
| L rostral anterior cingulate | 2.17 | 0.58 | 2.09 | 0.69 | 0.80 | 0.29 | 0.40 |
| L rostral middle frontal | 13.38 | 2.57 | 12.40 | 2.48 | 2.81 | **0.01** | 0.18 |
| L superior frontal | 19.87 | 3.47 | 18.80 | 3.22 | 2.61 | **0.02** | 0.20 |
| L superior parietal | 11.58 | 1.94 | 11.55 | 2.29 | 0.42 | 0.36 | 0.40 |
| L superior temporal | 10.70 | 1.77 | 10.10 | 1.70 | 2.44 | **0.02** | 0.20 |
| L supramarginal | 9.52 | 1.79 | 9.35 | 1.77 | 0.68 | 0.31 | 0.40 |
| L frontal pole | 0.87 | 0.16 | 0.85 | 0.18 | 0.43 | 0.36 | 0.40 |
| L temporal pole | 2.25 | 0.48 | 2.12 | 0.56 | 2.29 | **0.03** | 0.20 |
| L transverse temporal | 1.05 | 0.31 | 0.96 | 0.32 | 1.19 | 0.19 | 0.40 |
| L insula | 6.40 | 1.14 | 6.32 | 1.34 | 1.13 | 0.21 | 0.40 |

*R: right, L:left, std:standard deviation, p-val uncor: p-values uncorrected, p-val corr: p-values corrected by FDR. In bold corrected and uncorrected p-values <0.05.*

**Table 8.** Results of the surface area contrast between Class I and Class II-III for the subgroup of patients with CSF/Blood tests (linear regression analysis)

| **Structures** | **Control mean** | **Control std** | **Patient mean** | **Patient std** | **t stat** | **p-val uncor** | **p-val corr** |
| --- | --- | --- | --- | --- | --- | --- | --- |
| R caudal anterior cingulate | 6.41 | 0.97 | 6.16 | 1.08 | 1.43 | 0.14 | 0.39 |
| R caudal middle frontal | 19.37 | 3.83 | 19.04 | 3.97 | -0.56 | 0.34 | 0.39 |
| R cuneus | 15.14 | 2.74 | 15.02 | 3.12 | -0.93 | 0.25 | 0.39 |
| R enthorinal | 4.02 | 0.81 | 3.99 | 0.73 | 0.43 | 0.36 | 0.39 |
| R fusiform | 27.39 | 4.85 | 26.65 | 4.72 | -0.72 | 0.30 | 0.39 |
| R inferiorp arietal | 48.81 | 9.41 | 46.25 | 10.39 | 1.08 | 0.22 | 0.39 |
| R inferior temporal | 28.90 | 5.73 | 28.08 | 6.73 | -0.54 | 0.34 | 0.39 |
| R isthmus cingulate | 9.13 | 1.55 | 9.06 | 1.57 | -0.37 | 0.37 | 0.39 |
| R lateral occipital | 46.23 | 8.87 | 45.57 | 9.29 | -2.77 | **0.01** | 0.39 |
| R lateral orbitofrontal | 25.01 | 3.80 | 24.27 | 4.72 | 0.18 | 0.39 | 0.39 |
| R lingual | 29.96 | 6.21 | 29.43 | 5.09 | -1.19 | 0.19 | 0.39 |
| R medial orbitofrontal | 18.52 | 2.35 | 18.37 | 2.83 | -0.25 | 0.38 | 0.39 |
| R middle temporal | 30.83 | 4.99 | 29.79 | 6.02 | 0.17 | 0.39 | 0.39 |
| R parahippocampal | 6.19 | 1.10 | 6.12 | 1.04 | 0.05 | 0.39 | 0.39 |
| R paracentral | 14.98 | 2.06 | 14.22 | 2.01 | 1.13 | 0.21 | 0.39 |
| R pars opercularis | 12.08 | 2.00 | 11.66 | 1.95 | -0.20 | 0.39 | 0.39 |
| R pars orbitalis | 7.71 | 1.10 | 7.36 | 1.08 | 0.69 | 0.31 | 0.39 |
| R pars triangularis | 14.13 | 2.61 | 13.40 | 2.06 | 0.43 | 0.36 | 0.39 |
| R pericalcarine | 14.77 | 3.04 | 14.55 | 3.28 | -0.93 | 0.25 | 0.39 |
| R postcentral | 37.52 | 4.95 | 35.41 | 3.98 | 1.85 | 0.07 | 0.39 |
| R posterior cingulate | 11.15 | 1.84 | 10.84 | 1.47 | 0.59 | 0.33 | 0.39 |
| R precentral | 47.16 | 7.26 | 45.11 | 6.80 | 0.09 | 0.39 | 0.39 |
| R precuneus | 37.05 | 6.58 | 36.73 | 6.58 | -0.91 | 0.26 | 0.39 |
| R rostral anterior cingulate | 5.40 | 0.94 | 5.12 | 1.01 | 1.49 | 0.13 | 0.39 |
| R rostral middle frontal | 55.62 | 10.07 | 54.35 | 11.33 | -1.02 | 0.23 | 0.39 |
| R superior frontal | 65.74 | 11.00 | 63.84 | 12.24 | -0.91 | 0.26 | 0.39 |
| R superior parietal | 49.80 | 7.11 | 48.54 | 8.37 | 0.68 | 0.31 | 0.39 |
| R superior temporal | 34.23 | 5.83 | 32.50 | 4.51 | 0.77 | 0.29 | 0.39 |
| R supramarginal | 32.75 | 4.73 | 31.68 | 4.97 | 1.12 | 0.21 | 0.39 |
| R frontal pole | 2.95 | 0.43 | 2.87 | 0.51 | 0.19 | 0.39 | 0.39 |
| R temporal pole | 4.73 | 1.50 | 4.58 | 0.68 | 0.16 | 0.39 | 0.39 |
| R transverse temporal | 3.03 | 0.58 | 2.90 | 0.66 | 0.70 | 0.31 | 0.39 |
| R insula | 22.51 | 2.81 | 22.08 | 2.78 | 0.04 | 0.39 | 0.39 |
| L caudal anterior cingulate | 5.94 | 1.26 | 5.77 | 1.39 | -0.09 | 0.39 | 0.39 |
| L caudal middle frontal | 20.66 | 4.67 | 19.86 | 5.40 | -1.56 | 0.12 | 0.39 |
| L cuneus | 14.30 | 2.67 | 13.82 | 2.79 | 1.23 | 0.19 | 0.39 |
| L enthorinal | 4.49 | 0.98 | 4.46 | 1.04 | -0.46 | 0.35 | 0.39 |
| L fusiform | 28.91 | 4.43 | 27.50 | 4.71 | 0.23 | 0.38 | 0.39 |
| L inferior parietal | 40.75 | 7.23 | 39.40 | 8.20 | 1.19 | 0.19 | 0.39 |
| L inferior temporal | 30.30 | 6.17 | 29.40 | 8.17 | -0.27 | 0.38 | 0.39 |
| L isthmus cingulate | 10.21 | 1.57 | 9.86 | 1.79 | 0.12 | 0.39 | 0.39 |
| L lateral occipital | 47.03 | 7.57 | 45.48 | 7.87 | 0.21 | 0.39 | 0.39 |
| L lateral orbitofrontal | 25.66 | 3.57 | 25.09 | 4.30 | -0.48 | 0.35 | 0.39 |
| L lingual | 29.18 | 4.89 | 28.98 | 4.64 | -2.01 | 0.06 | 0.39 |
| L medial orbitofrontal | 17.95 | 2.14 | 17.56 | 2.64 | -0.31 | 0.38 | 0.39 |
| L middle temporal | 28.36 | 5.04 | 26.65 | 6.06 | 1.54 | 0.12 | 0.39 |
| L parahippocampal | 6.35 | 0.63 | 6.21 | 0.65 | 0.49 | 0.35 | 0.39 |
| L paracentral | 13.58 | 2.45 | 13.60 | 3.07 | -1.45 | 0.14 | 0.39 |
| L pars opercularis | 15.05 | 2.93 | 14.64 | 3.65 | -0.42 | 0.36 | 0.39 |
| L pars orbitalis | 6.54 | 1.06 | 6.11 | 1.18 | 1.44 | 0.14 | 0.39 |
| L pars triangularis | 12.71 | 2.29 | 12.17 | 2.32 | 0.14 | 0.39 | 0.39 |
| L pericalcarine | 13.45 | 2.49 | 13.29 | 2.68 | -0.56 | 0.34 | 0.39 |
| L postcentral | 38.42 | 4.62 | 36.95 | 4.50 | 0.60 | 0.33 | 0.39 |
| L posterior cingulate | 11.29 | 1.86 | 10.77 | 1.84 | 1.26 | 0.18 | 0.39 |
| L precentral | 46.01 | 7.48 | 44.79 | 8.09 | -1.17 | 0.20 | 0.39 |
| L precuneus | 36.86 | 6.16 | 35.58 | 6.52 | 0.64 | 0.32 | 0.39 |
| L rostral anterior cingulate | 7.18 | 1.82 | 6.93 | 1.64 | 0.05 | 0.39 | 0.39 |
| L rostral middle frontal | 52.42 | 9.67 | 48.42 | 10.61 | 1.75 | 0.09 | 0.39 |
| L superior frontal | 68.33 | 11.35 | 65.20 | 12.34 | 0.15 | 0.39 | 0.39 |
| L superior parietal | 50.61 | 7.84 | 49.42 | 9.10 | 0.80 | 0.28 | 0.39 |
| L superior temporal | 36.81 | 5.28 | 35.03 | 5.70 | 0.90 | 0.26 | 0.39 |
| L supramarginal | 36.57 | 6.42 | 35.64 | 6.42 | -0.53 | 0.34 | 0.39 |
| L frontal pole | 2.37 | 0.31 | 2.29 | 0.36 | 0.24 | 0.38 | 0.39 |
| L temporal pole | 4.66 | 0.64 | 4.59 | 0.81 | -0.13 | 0.39 | 0.39 |
| L transverse temporal | 4.34 | 0.94 | 4.09 | 0.95 | 0.74 | 0.30 | 0.39 |
| L insula | 22.84 | 3.29 | 22.80 | 3.84 | -0.26 | 0.38 | 0.39 |

*R: right, L:left, std:standard deviation, p-val uncor: p-values uncorrected, p-val corr: p-values corrected by FDR. In bold corrected and uncorrected p-values <0.05.*

**Table 9.** Results of the cortical thickness contrast between Class I and Class II-III for the subgroup of patients with CSF/Blood tests (linear regression analysis)

| **Structures** | **Control**  **mean** | **Control std** | **Patient mean** | **Patient std** | **t stat** | **p-val**  **uncor** | **p-val**  **cor** |
| --- | --- | --- | --- | --- | --- | --- | --- |
| R caudal anterior cingulate | 2.39 | 0.18 | 2.41 | 0.23 | -0.14 | 0.39 | 0.40 |
| R caudal middle frontal | 2.38 | 0.25 | 2.35 | 0.27 | 0.64 | 0.32 | 0.40 |
| R cuneus | 1.86 | 0.20 | 1.87 | 0.24 | -0.57 | 0.33 | 0.40 |
| R enthorinal | 3.39 | 0.38 | 3.41 | 0.39 | 0.25 | 0.38 | 0.40 |
| R fusiform | 2.60 | 0.20 | 2.58 | 0.20 | 1.39 | 0.15 | 0.40 |
| R inferior parietal | 2.35 | 0.16 | 2.36 | 0.16 | -0.61 | 0.33 | 0.40 |
| R inferior temporal | 2.62 | 0.18 | 2.63 | 0.15 | -0.33 | 0.37 | 0.40 |
| R isthmus cingulate | 2.30 | 0.22 | 2.30 | 0.21 | 0.02 | 0.40 | 0.40 |
| R lateral occipital | 2.15 | 0.14 | 2.13 | 0.17 | 1.17 | 0.20 | 0.40 |
| R lateral orbitofrontal | 2.52 | 0.19 | 2.50 | 0.19 | -0.08 | 0.39 | 0.40 |
| R lingual | 1.99 | 0.15 | 1.95 | 0.18 | 0.98 | 0.24 | 0.40 |
| R medial orbitofrontal | 2.35 | 0.18 | 2.32 | 0.16 | 1.13 | 0.21 | 0.40 |
| R middle temporal | 2.59 | 0.21 | 2.64 | 0.23 | -1.00 | 0.24 | 0.40 |
| R parahippocampal | 2.63 | 0.25 | 2.61 | 0.25 | 0.35 | 0.37 | 0.40 |
| R paracentral | 2.32 | 0.23 | 2.29 | 0.28 | 0.68 | 0.31 | 0.40 |
| R pars opercularis | 2.42 | 0.21 | 2.43 | 0.21 | 0.00 | 0.40 | 0.40 |
| R pars orbitalis | 2.63 | 0.21 | 2.64 | 0.21 | -0.84 | 0.28 | 0.40 |
| R pars triangularis | 2.31 | 0.22 | 2.32 | 0.22 | -1.26 | 0.18 | 0.40 |
| R pericalcarine | 1.59 | 0.31 | 1.57 | 0.22 | 0.85 | 0.27 | 0.40 |
| R postcentral | 1.93 | 0.19 | 1.93 | 0.20 | -1.03 | 0.23 | 0.40 |
| R posterior cingulate | 2.35 | 0.24 | 2.31 | 0.28 | 1.19 | 0.19 | 0.40 |
| R precentral | 2.37 | 0.29 | 2.34 | 0.33 | 1.24 | 0.18 | 0.40 |
| R precuneus | 2.28 | 0.17 | 2.28 | 0.18 | -0.80 | 0.29 | 0.40 |
| R rostral anterior cingulate | 2.73 | 0.23 | 2.69 | 0.23 | 1.43 | 0.14 | 0.40 |
| R rostral middle frontal | 2.23 | 0.19 | 2.24 | 0.19 | -1.79 | 0.08 | 0.40 |
| R superior frontal | 2.59 | 0.23 | 2.56 | 0.27 | 0.22 | 0.39 | 0.40 |
| R superior parietal | 2.09 | 0.16 | 2.09 | 0.19 | -0.62 | 0.32 | 0.40 |
| R superior temporal | 2.56 | 0.22 | 2.54 | 0.27 | 0.94 | 0.25 | 0.40 |
| R supramarginal | 2.34 | 0.19 | 2.37 | 0.21 | -1.40 | 0.15 | 0.40 |
| R frontal pole | 2.54 | 0.30 | 2.57 | 0.26 | 0.04 | 0.39 | 0.40 |
| R temporal pole | 3.50 | 0.38 | 3.40 | 0.43 | 1.57 | 0.12 | 0.40 |
| R transverse temporal | 2.28 | 0.34 | 2.25 | 0.38 | 1.09 | 0.22 | 0.40 |
| R insula | 2.80 | 0.26 | 2.79 | 0.30 | 1.28 | 0.17 | 0.40 |
| L caudal anterior cingulate | 2.61 | 0.25 | 2.62 | 0.26 | -0.14 | 0.39 | 0.40 |
| L caudal middle frontal | 2.42 | 0.22 | 2.40 | 0.25 | 1.10 | 0.21 | 0.40 |
| L cuneus | 1.80 | 0.23 | 1.82 | 0.30 | -1.27 | 0.17 | 0.40 |
| L enthorinal | 3.31 | 0.33 | 3.32 | 0.35 | 0.15 | 0.39 | 0.40 |
| L fusiform | 2.60 | 0.23 | 2.58 | 0.26 | 1.14 | 0.20 | 0.40 |
| L inferior parietal | 2.33 | 0.21 | 2.33 | 0.25 | -0.52 | 0.34 | 0.40 |
| L inferior temporal | 2.64 | 0.19 | 2.64 | 0.23 | 0.77 | 0.29 | 0.40 |
| L isthmus cingulate | 2.24 | 0.24 | 2.26 | 0.26 | -0.91 | 0.26 | 0.40 |
| L lateral occipital | 2.09 | 0.16 | 2.07 | 0.20 | 0.88 | 0.27 | 0.40 |
| L lateral orbitofrontal | 2.53 | 0.22 | 2.52 | 0.26 | 0.30 | 0.38 | 0.40 |
| L lingual | 1.93 | 0.19 | 1.95 | 0.23 | -1.25 | 0.18 | 0.40 |
| L medial orbitofrontal | 2.37 | 0.20 | 2.33 | 0.20 | 1.43 | 0.14 | 0.40 |
| L middle temporal | 2.63 | 0.20 | 2.66 | 0.24 | -0.29 | 0.38 | 0.40 |
| L parahippocampal | 2.75 | 0.29 | 2.71 | 0.30 | 0.57 | 0.33 | 0.40 |
| L paracentral | 2.27 | 0.25 | 2.28 | 0.29 | -0.75 | 0.30 | 0.40 |
| L pars opercularis | 2.41 | 0.22 | 2.39 | 0.26 | 0.57 | 0.33 | 0.40 |
| L pars orbitalis | 2.57 | 0.23 | 2.55 | 0.23 | 0.20 | 0.39 | 0.40 |
| L pars triangularis | 2.32 | 0.27 | 2.33 | 0.30 | 0.15 | 0.39 | 0.40 |
| L pericalcarine | 1.57 | 0.22 | 1.58 | 0.30 | -1.02 | 0.23 | 0.40 |
| L postcentral | 1.96 | 0.18 | 1.98 | 0.21 | -2.07 | 0.05 | 0.40 |
| L posterior cingulate | 2.35 | 0.23 | 2.35 | 0.26 | 0.72 | 0.30 | 0.40 |
| L precentral | 2.40 | 0.25 | 2.38 | 0.29 | 0.83 | 0.28 | 0.40 |
| L precuneus | 2.24 | 0.21 | 2.24 | 0.26 | -0.10 | 0.39 | 0.40 |
| L rostral anterior cingulate | 2.73 | 0.26 | 2.69 | 0.30 | 1.23 | 0.18 | 0.40 |
| L rostral middle frontal | 2.29 | 0.18 | 2.30 | 0.20 | -1.15 | 0.20 | 0.40 |
| L superior frontal | 2.60 | 0.25 | 2.58 | 0.31 | 0.10 | 0.39 | 0.40 |
| L superior parietal | 2.10 | 0.17 | 2.11 | 0.21 | -2.24 | **0.04** | 0.40 |
| L superior temporal | 2.53 | 0.23 | 2.51 | 0.27 | 0.71 | 0.30 | 0.40 |
| L supramarginal | 2.35 | 0.20 | 2.37 | 0.26 | -1.96 | 0.06 | 0.40 |
| L frontal pole | 2.62 | 0.28 | 2.59 | 0.30 | 0.22 | 0.38 | 0.40 |
| L temporal pole | 3.32 | 0.43 | 3.23 | 0.49 | 1.84 | 0.08 | 0.40 |
| L transverse temporal | 2.21 | 0.32 | 2.17 | 0.34 | 0.94 | 0.25 | 0.40 |
| L insula | 2.83 | 0.28 | 2.80 | 0.34 | 0.24 | 0.38 | 0.40 |

*R: right, L:left, std:standard deviation, p-val uncor: p-values uncorrected, p-val corr: p-values corrected by FDR. In bold corrected and uncorrected p-values <0.05.*

**Table 10**. The significant associations between cortical GMV and clinical variables in the SARS-CoV-2 group.

| **Clinical variables** | **Brain Structures** | **Corr** | **pval** |
| --- | --- | --- | --- |
| CSF Leukocytes | Right lateral orbitofrontal | -0.83 | 0.03 |
|  | Left inferior temporal | -0.83 | 0.03 |
| CSF Lactate | Left inferior temporal | -0.85 | 0.02 |
|  | Left rostral middle frontal | -0.79 | 0.04 |
|  | Right lateral orbitofrontal | -0.77 | 0.04 |
| CSF Protein | Right lateral orbitofrontal | -0.84 | 0.01 |
|  | Left inferior temporal | -0.86 | 0.01 |
|  | Left fusiform | -0.85 | 0.01 |
|  | Right inferior parietal | -0.83 | 0.01 |
|  | Right medial orbitofrontal | -0.78 | 0.02 |
|  | Right superior temporal | -0.77 | 0.02 |
|  | Left lateral orbitofrontal | -0.78 | 0.02 |
|  | Left inferior parietal | -0.75 | 0.03 |
|  | Left rostral middle frontal | -0.73 | 0.03 |
|  | Left medial orbitofrontal | -0.73 | 0.03 |
|  | Right transverse temporal | -0.71 | 0.04 |
|  | Left paracentral | -0.69 | 0.04 |
|  | Right caudal middle frontal | -0.69 | 0.04 |
|  | Right fusiform | -0.68 | 0.04 |
|  | Right rostral middle frontal | -0.69 | 0.04 |
|  | Left lateral occipital | -0.68 | 0.04 |
|  | Left superior temporal | -0.67 | 0.04 |
|  | Right superior frontal | -0.66 | 0.04 |
| CSF/blood-albumin ratio | Right lateral orbitofrontal | -0.93 | 0.01 |
|  | Left inferior temporal | -0.88 | 0.01 |
|  | Right inferior parietal | -0.86 | 0.01 |
|  | Right caudal middle frontal | -0.84 | 0.01 |
|  | Left medial orbitofrontal | -0.83 | 0.01 |
|  | Right medial orbitofrontal | -0.81 | 0.01 |
|  | Left inferior parietal | -0.81 | 0.01 |
|  | Left rostral middle frontal | -0.80 | 0.01 |
|  | Left paracentral | 0.79 | 0.01 |
|  | Left rostral anterior cingulate | -0.79 | 0.01 |
|  | Left fusiform | -0.78 | 0.02 |
|  | Right fusiform | -0.74 | 0.03 |
|  | Right rostral middle frontal | -0.73 | 0.03 |
|  | Right superior frontal | -0.71 | 0.04 |
|  | Right superior temporal | -0.70 | 0.04 |
| CSF EN-RAGE | Right caudal middle frontal | -0.91 | 0.01 |
|  | Left rostral middle frontal | -0.85 | 0.01 |
|  | Right lateral orbitofrontal | -0.86 | 0.01 |
|  | Right rostral middle frontal | -0.79 | 0.01 |
|  | Left inferior temporal | -0.80 | 0.01 |
|  | Right fusiform | -0.79 | 0.01 |
|  | Left medial orbitofrontal | -0.79 | 0.01 |
|  | Left rostral anterior cingulate | -0.78 | 0.01 |
|  | Left paracentral | 0.77 | 0.01 |
|  | Right inferior temporal | -0.74 | 0.02 |
|  | Right superior frontal | -0.74 | 0.02 |
|  | Left caudal middle frontal | -0.72 | 0.02 |
|  | Right middle temporal | -0.68 | 0.04 |
|  | Right medial orbitofrontal | -0.68 | 0.04 |
|  | Left superior frontal | -0.66 | 0.04 |
|  | Right inferior parietal | -0.67 | 0.04 |
|  | Left fusiform | -0.66 | 0.04 |

*Partial correlation value and their corrected p-value (FDR) are shown. CSF= Cerebrospinal fluid, EN-RAGE= extracellular receptor for advanced glycation end-products binding protein, pval= p values corrected by FDR, Corr= correlation, L= left hemisphere, R= right hemisphere.*

**Table 11**. The significant associations between regional CTh and clinical variables in the SARS-CoV-2 group.

| Clinical variables | Brain Structures | Corr | pval |
| --- | --- | --- | --- |
| CSF/blood albumin-ratio | Left lateral orbitofrontal | 0.84 | 0.03 |
|  | Left pars triangularis | 0.84 | 0.03 |
|  | Left rostral anterior cingulate | -0.81 | 0.03 |
|  | Left postcentral | 0.79 | 0.04 |
|  | Right paracentral | 0.80 | 0.04 |
|  | Right caudal middle frontal | -0.79 | 0.04 |
| CSF EN-RAGE | Left pars triangularis | 0.88 | 0.01 |

*Partial correlation value and their corrected p-value (FDR) are shown. CSF=Cerebrospinal fluid, EN-RAGE=extracellular receptor for advanced glycation end-products binding protein, pval= p values corrected by FDR, Corr= correlation, L= left hemisphere, R= right hemisphere.*

**References**

Bernardi, S., Bossi, F., Toffoli, B., & Fabris, B. (2016). Roles and Clinical Applications of OPG and TRAIL as Biomarkers in Cardiovascular Disease. *BioMed Research International*, *2016*. https://doi.org/10.1155/2016/1752854

Drago, V., Babiloni, C., Bartr??s-Faz, D., Caroli, A., Bosch, B., Hensch, T., Didic, M., Klafki, H. W., Pievani, M., Jovicich, J., Venturi, L., Spitzer, P., Vecchio, F., Schoenknecht, P., Wiltfang, J., Redolfi, A., Forloni, G., Blin, O., Irving, E., … Frisoni, G. B. (2011). Disease tracking markers for Alzheimer’s disease at the prodromal (MCI) stage. *Advances in Alzheimer’s Disease*, *2*, 331–371. https://doi.org/10.3233/978-1-60750-793-2-331

Hudson, B. I., & Lippman, M. E. (2018). Targeting RAGE Signaling in Inflammatory Disease. *Https://Doi.Org/10.1146/Annurev-Med-041316-085215*, *69*, 349–364. https://doi.org/10.1146/ANNUREV-MED-041316-085215

Huy, N. T., Thao, N. T. H., Diep, D. T. N., Kikuchi, M., Zamora, J., & Hirayama, K. (2010). 1Cerebrospinal fluid lactate concentration to distinguish bacterial from aseptic meningitis: A systemic review and meta-analysis. *Critical Care*, *14*(6), 1–15. https://doi.org/10.1186/CC9395/FIGURES/6

Jarius, S., Pache, F., Körtvelyessy, P., Jelčić, I., Stettner, M., Franciotta, D., Keller, E., Neumann, B., Ringelstein, M., Senel, M., Regeniter, A., Kalantzis, R., Willms, J. F., Berthele, A., Busch, M., Capobianco, M., Eisele, A., Reichen, I., Dersch, R., … Wildemann, B. (2022). Cerebrospinal fluid findings in COVID-19: a multicenter study of 150 lumbar punctures in 127 patients. *Journal of Neuroinflammation*, *19*(1), 1–33. https://doi.org/10.1186/S12974-021-02339-0/FIGURES/9

Josien, R., Wong, B. R., Li, H.-L., Steinman, R. M., & Choi, Y. (1999). TRANCE, a TNF Family Member, Is Differentially Expressed on T Cell Subsets and Induces Cytokine Production in Dendritic Cells. *The Journal of Immunology*, *162*(5).

Kroksveen, A. C., Opsahl, J. A., Aye, T. T., Ulvik, R. J., & Berven, F. S. (2011). Proteomics of human cerebrospinal fluid: Discovery and verification of biomarker candidates in neurodegenerative diseases using quantitative proteomics. *Journal of Proteomics*, *74*(4), 371–388. https://doi.org/10.1016/J.JPROT.2010.11.010

Meijer, B., Gearry, R. B., & Day, A. S. (2012). The role of s100a12 as a systemic marker of inflammation. *International Journal of Inflammation*, *2012*. https://doi.org/10.1155/2012/907078

Moore, B. A., & Barnett, J. E. (2016). Cerebrospinal Fluid. *Case Studies in Clinical Psychological Science: Bridging the Gap from Science to Practice*, *August*, 1–7. https://doi.org/10.1093/MED/9780199937837.003.0145

Shahan, B., Choi, E. Y., & Nieves, G. (2021). Cerebrospinal Fluid Analysis. *American Family Physician*, *103*(7), 422–428. https://www.aafp.org/pubs/afp/issues/2021/0401/p422.html

*TNFRSF11B TNF receptor superfamily member 11b [Homo sapiens (human)] - Gene - NCBI*. (n.d.). Retrieved September 28, 2022, from https://www.ncbi.nlm.nih.gov/gene/4982
